# Efficient in vivo genome editing prevents hypertrophic cardiomyopathy in mice

**DOI:** 10.1038/s41591-022-02190-7

**Published:** 2023-02-16

**Authors:** Daniel Reichart, Gregory A. Newby, Hiroko Wakimoto, Mingyue Lun, Joshua M. Gorham, Justin J. Curran, Aditya Raguram, Daniel M. DeLaughter, David A. Conner, Júlia D. C. Marsiglia, Sajeev Kohli, Lukas Chmatal, David C. Page, Nerea Zabaleta, Luk Vandenberghe, David R. Liu, Jonathan G. Seidman, Christine Seidman

**Affiliations:** 1 Department of Genetics, Harvard Medical School, Boston, MA USA; 2 Department of Medicine I, University Hospital, LMU Munich, Munich, Germany; 3 Merkin Institute of Transformative Technologies in Healthcare, Broad Institute of Harvard and MIT, Cambridge, MA USA; 4 Department of Chemistry and Chemical Biology, Harvard University, Cambridge, MA USA; 5 Howard Hughes Medical Institute, Chevy Chase, MD USA; 6 Whitehead Institute, Cambridge, MA USA; 7 Department of Biology, Massachusetts Institute of Technology, Cambridge, MA USA; 8 Grousbeck Gene Therapy Center, Schepens Eye Research Institute, Mass Eye and Ear, Boston, MA USA; 9 Ocular Genomics Institute, Department of Ophthalmology, Harvard Medical School, Boston, MA USA; 10 Harvard Stem Cell Institute, Harvard University, Cambridge, MA USA; 11 Cardiovascular Division, Brigham and Women’s Hospital, Boston, MA USA

**Keywords:** Mutation, Cardiac hypertrophy

## Abstract

Dominant missense pathogenic variants in cardiac myosin heavy chain cause hypertrophic cardiomyopathy (HCM), a currently incurable disorder that increases risk for stroke, heart failure and sudden cardiac death. In this study, we assessed two different genetic therapies—an adenine base editor (ABE8e) and a potent Cas9 nuclease delivered by AAV9—to prevent disease in mice carrying the heterozygous HCM pathogenic variant myosin R403Q. One dose of dual-AAV9 vectors, each carrying one half of RNA-guided ABE8e, corrected the pathogenic variant in ≥70% of ventricular cardiomyocytes and maintained durable, normal cardiac structure and function. An additional dose provided more editing in the atria but also increased bystander editing. AAV9 delivery of RNA-guided Cas9 nuclease effectively inactivated the pathogenic allele, albeit with dose-dependent toxicities, necessitating a narrow therapeutic window to maintain health. These preclinical studies demonstrate considerable potential for single-dose genetic therapies to correct or silence pathogenic variants and prevent the development of HCM.

## Abstract (web summary)

Two approaches using an adenine base editor and a Cas9 nuclease prevented the development of hypertrophic cardiomyopathy in mice carrying a pathogenic mutation on the *Myh6* gene, highlighting the potential of single-dose genetic therapies for the treatment of cardiac disease.

## Main

Hypertrophic cardiomyopathy (HCM), a primary myocardial disorder that is estimated to occur in one in 500 individuals, causes left ventricular hypertrophy (LVH) with diminished LV volumes and increases myocardial fibrosis^1,2^. HCM arises from dominant variants that alter sarcomere components, including the contractile protein cardiac myosin heavy chain^3^. Pathogenic myosin variants produce symptomatic HCM early in adulthood with high rates of adverse outcomes, including heart failure, arrhythmias, stroke and sudden cardiac death^4^. Although medications and devices reduce arrhythmias and sudden cardiac death, treatments cannot prevent heart failure, the leading cause for cardiac transplantation and premature death in patients with HCM^4^.

Discovery of the molecular basis for HCM enables early, gene-based diagnosis with longitudinal assessments of clinical manifestations. Childhood carriers of pathogenic variants often demonstrate a latency period of variable duration when LVH and symptoms are absent^5–7^, whereas adults demonstrate LVH, the diagnostic criteria for HCM. Although factors that influence disease emergence remain unknown, this natural history of disease indicates a window of opportunity for potential interventions to limit or prevent disease.

Nucleic acid therapies are potential medicines for genetic diseases. RNA-based treatments (small interfering or hairpin RNAs and antisense oligonucleotides) are effective but short-lived, necessitating recurrent administrations for long-term efficacy^8^. Alternatively, permanent DNA modification can be achieved with genome editors delivered by vectors, such as adeno-associated virus (AAV)^9,10^.

We previously engineered a heterozygous, pathogenic myosin variant orthologous to the human p.R403Q allele into the mouse *Myh6* gene^11^, which encodes the predominant murine cardiac myosin isoform. 129SvEv mice harboring one R403Q variant allele recapitulate key features of human HCM, including LVH, altered chamber volumes and myocardial fibrosis^11,12^.

Using R403Q mice, we showed that intra-thoracic delivery of a cardiotropic vector—AAV9-encoding small interfering RNAs (siRNAs) expressed under the control of the cardiomyocyte-specific chicken troponin T (*Tnnt2*) promoter^13^—selectively silenced the mutant transcript^14^. A 25% reduction of mutant transcripts levels was sufficient to prevent the emergence of LVH for 6 months, after which durability waned.

We recently studied base editor therapies in transgenic mice expressing a pathogenic lamin variant^15^ that causes human progeria^16^ with cardiovascular disease. Using dual-AAV9 vectors, each containing one half of a split-intein adenine base editor (ABE)^17^ to overcome the inadequate packaging limit (~4.7 kb) of AAV9 vectors for full-length base editors, we achieved modest genomic editing (~20–30%) in cardiovascular tissues that enabled twofold-longer survival than untreated mice^17^.

Capitalizing on these advances, we and authors of the accompanying manuscript (Chai et al.) evaluated different strategies to edit or silence the HCM allele in R403Q mice. We report genomic editing corrected over 70% of mutant transcripts, with tolerable frequencies of edited bystander nucleotides (for example, proximal to the targeted adenosine residue). Treatments were well tolerated and prevented functional, histopathological, molecular phenotypes of HCM that emerge slowly or rapidly in mouse models. We also assessed permanently silencing of the R403Q allele, using *Staphylococcus aureus* Cas9, encoded within a single AAV9 vector. This strategy efficiently edited cardiomyocytes and prevented LVH but with dose-dependent deleterious effects on contractile function, indicating a narrow therapeutic window.

## Results

### Design of a dual-AAV9-encoded base editor and protospacer

**Extended Data Fig. 1:**
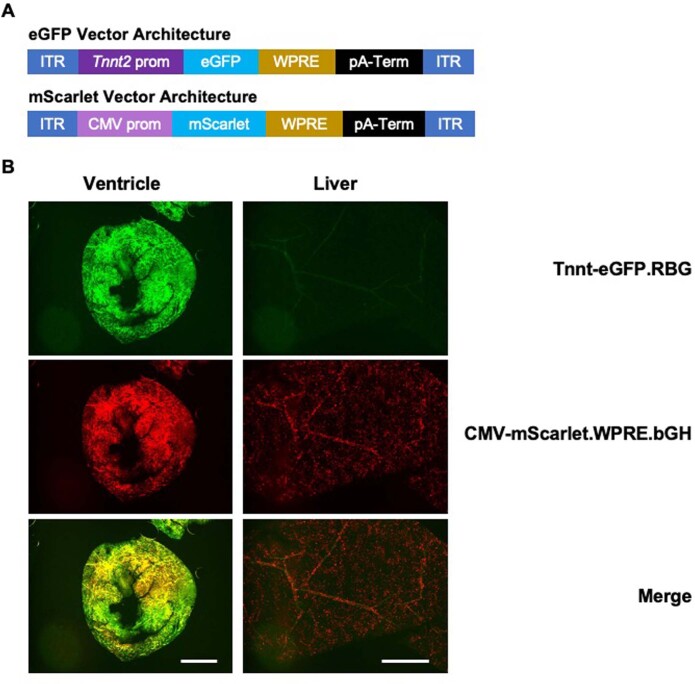
AAV9 vectors used to assess cardiotropic co-expression of fluorescent marker genes. **A)** Schematics of AAV9 genomes containing fluorescent reporter genes (not to scale) used to assess dual transduction. ITR: AAV9 inverted terminal repeats. *Tnnt2* prom: cardiomyocyte specific troponin T promoter. CMV prom: Cytomegalovirus promoter, a strong, constitutive promoter. WPRE: Woodchuck hepatitis virus post-transcriptional regulatory element, used to stabilize transcripts. pA-Term: polyadenylation signal and transcriptional terminator. **B)** Fluorescent micrographs of ventricular section (short axis view) and liver from a WT mouse injected with 3×10^13^ vg/kg AAV2/9-Tnnt2-eGFP.RBG (Tnnt2-eGFP.RBG) and AAV2/9-CMV-mScarlet.WPRE.bGH (CMV-mScarlet.WPRE.bGH) and imaged with a fluorescence microscope. Scale bar: 1 mm. Imaging revealed co-expression of green fluorescent protein (top panel) delivered by AAV9-Tnnt2-eGFP.RBG and red fluorescent protein (middle panel) delivered by AAV9-CMV-mScarlet.WPRE.bGH are co-localized (yellow signal, lower panel) in cardiac cells. Representative images are shown from two technical replicates.

We used a split-intein design, requiring co-transduction of two AAV vectors, each expressing half of the base editor, and trans-splicing reconstitution of the full-length base editor within cells^18,19^. To assess toxicity and efficiency of dual-AAV9 infection of cardiomyocytes, we performed pilot experiments using two AAV9 viruses expressing fluorescent markers eGFP under the *Tnnt2* promoter^14^ or mScarlet driven by the broadly expressed *CMV* promoter (Extended Data Fig. 1a). Wild-type (WT) mice receiving intra-thoracic injection of 3 × 10^13^ vector genomes per kilogram (vg kg^−1^) for each vector had normal vitality and survival. Tissues harvested 3 weeks after injection showed normal histology and prominent dual fluorescence in the heart but not liver (Extended Data Fig. 1b).

**Fig. 1:**
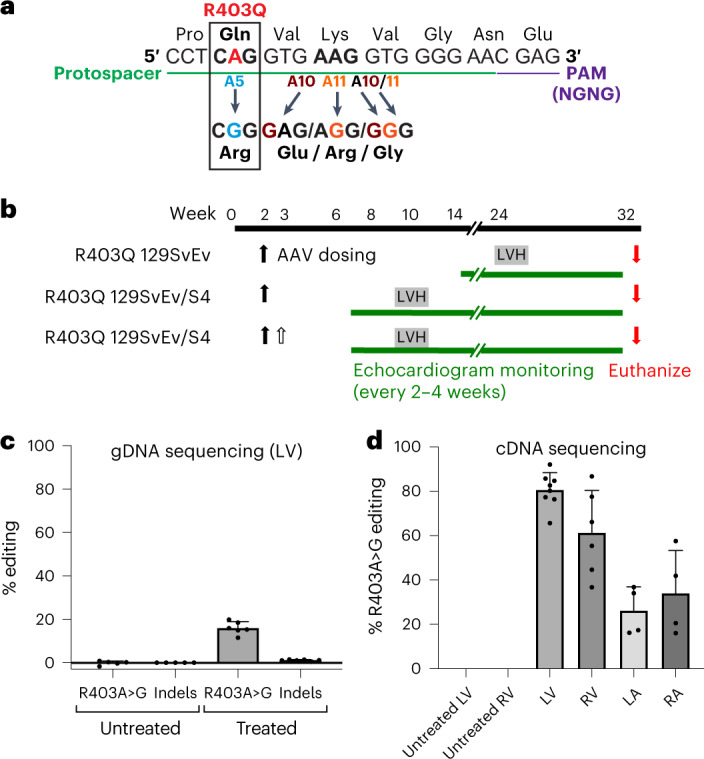
Base editing the pathogenic variant R403Q mediated by injection of dual-AAV9 ABE8e. **a**, Schematic of the genomic sequence surrounding the HCM R403Q pathogenic variant. The ABE protospacer (green line) and PAM (purple line) are underlined. The pathogenic variant R403Q (numbered according to the human *MYH7* amino acid residue) is shown with flanking amino acid residues (N, amino terminus; C, carboxyl terminus). R403Q is located at position A5 (blue), and potential bystander edits are at positions 10 (brown) and 11 (orange), numbered from the 5′ end of the protospacer. Bystander editing at each position (arrows) would encode missense amino acids. **b**, Schematic of the experimental design depicting AAV9 delivery and subsequent evaluation of disease. Dual-AAV9 ABE8e or single AAV9-Cas9 were injected (solid filled arrow, first dose; unfilled arrow, second dose) into R403Q-129SvEv or R403Q-129SvEv/S4 mice at postnatal weeks 2–3. All R403Q-129SvEv/S4 mice consistently develop LVH by 8–10 weeks (gray box), whereas only male R403Q-129SvEv mice show LVH at 20–25 weeks (blue box). Cardiac morphology and function were assessed by echocardiography at 2–4-week intervals. **c**, Editing efficiency (%) of the targeted pathogenic variant R403Q was based on high-throughput sequencing gDNA from all LV cells, including cardiomyocytes, fibroblasts, macrophages and endothelial cells, after a single dose of dual-AAV9 ABE8e injection (*n* = 6 males) or in untreated R403Q mice (*n* = 5 males), quantified by CRISPResso2. gDNA editing efficiency was calculated as: WT nucleotide percentage minus 50% (to measure the editing beyond the heterozygous baseline) and then divided by 50% (to determine the percentage of observed editing out of the theoretical maximum). **d**, Editing efficiency of the targeted R403Q allele after a single AAV9 ABE8e injection was assessed by sequencing *Myh6* cDNA derived from RNA extracted from the LV (*n* = 5 males, 3 females), RV (*n* = 4 males, 2 females), LA (*n* = 2 males, 2 females) and RA (*n* = 2 males, 2 females) and in five untreated LVs and RVs from male mice. RNA editing efficiency was calculated as: WT nucleotide percentage minus 50% and then divided by 50%. Data are presented as mean values ± s.d. Source data

**Extended Data Fig. 2:**
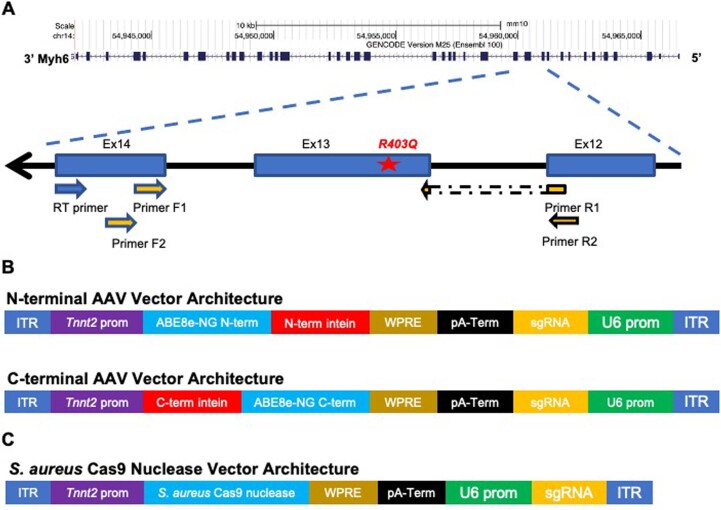
Schematic of *Myh6* gene and AAV vector genomes. **A)** Schematic of *Myh6* gene indicating the location of the pathogenic variant R403Q (numbered according to the human amino acid residues) in exon 13 and the targeted *Myh6* mRNA region that was amplified to assess editing (Methods). The locations of reverse transcriptase primer (RT-primer) and PCR primer pairs 1 and 2 are indicated. **B)** Schematics of dual AAV vector genomes (not to scale) that encode an amino-terminal fragment (N-term) and carboxyl-terminal fragment (C-term) that form base editors after protein splicing. ITR: inverted terminal repeats. *Tnnt2* Prom: the cardiomyocyte-specific troponin T promoter. WPRE: Woodchuck hepatitis virus post-transcriptional regulatory element, used to stabilize transcripts. pA-Term: polyadenylation signal and transcriptional terminator. sgRNA: single guide RNA. U6 prom: the human Pol III promoter that efficiently transcribes guide RNAs. **C)** Schematic of the AAV vector genome used for Cas9 nuclease delivery (not to scale). ITR: Inverted terminal repeats. *Tnnt2* prom: the cardiomyocyte specific troponin T promoter. WPRE: Woodchuck hepatitis virus post-transcriptional regulatory element, used to stabilize transcripts. pA-Term: polyadenylation signal and transcriptional terminator. U6 prom: the human Pol III promoter that efficiently transcribes guide RNAs. sgRNA: single guide RNA.

The pathogenic variant R403Q results from a G>A transition (Extended Data Fig. 2a), an ABE target. We used ABE8e, a particularly active deaminase^20^, and designed a protospacer that places the R403Q variant at position A5 (counting from the PAM-distal end of the protospacer) (Fig. 1a). This single guide RNA (sgRNA) uses a nearby ‘CGAG’ PAM, which is recognized efficiently by a *Streptococcus pyogenes* Cas9 variant SpCas9-NG^18,19^ that we selected for base editing. We introduced each half of the base editor into dual-AAV9s under the control of the *Tnnt2* promoter along with an identical sgRNA to direct the ABE8e (Extended Data Fig. 2b).

The *Tnnt2* promoter should express the dual AAV cargos at high levels only in cardiomyocytes and, thereby, exclude base editing in other cell lineages. We confirmed cardiomyocyte-enriched expression for the split-intein ABE cargo using single-nucleus RNA sequencing (snRNA-seq) of all cardiac chambers from four treated mice (Supplementary Table 1). *Tnnt2-*promoted AAV9 encoded transcripts in 55% ± 14% of left ventricle (LV) cardiomyocytes, 41% ± 10% of right ventricle (RV) cardiomyocytes, 21% ± 9% of left atria (LA) cardiomyocytes and 35% ± 11% of right atria (RA) cardiomyocytes. Although significantly fewer atrial than ventricular cardiomyocytes expressed AAV9-encoded cargo (*P* = 0.001), *Tnnt2-*promoted transcripts in seven other LV cell lineages were much lower (mean expression = 2.1% ± 1.7%).

### Base editing of two HCM mouse models and in vivo monitoring

We targeted the R403Q variant in two mouse lines (Fig. 1b). R403Q-129SvEv male mice develop LVH at 20–25 weeks of age^11,12,21^, thereby modeling insidious onset of HCM in human patients. R403Q-129SvEv/S4 hybrid mice, produced from a fortuitous R403Q-129SvEv and 129S4 cross, develop rapid onset of LVH in males and females by 8–10 weeks of age, thereby modeling patients with fulminant HCM^22^.

R403Q-129SvEv mice, age 10–13 days, were injected in the thoracic cavity^14,23^ with one dose of dual-AAV9 carrying ABE8e (1.25 × 10^13^ vg kg^−1^ of each vector; Fig. 1b). As initial analyses of LVs from two mice at 3 weeks of age confirmed editing, cardiac function was studied by echocardiography until sacrifice at age 32–34 weeks in treated and untreated R403Q and WT mice. Editing efficiency in bulk genomic DNA (gDNA) and bulk RNA was calculated as the percentage (%) of the WT G nucleotide minus 50% (to measure editing beyond the heterozygous baseline) and then divided by 50% (to determine the percentage of observed editing beyond the theoretical maximum). For heterozygous variants, this measure of editing efficiency corresponds to the total percentage of edited cells.

High-throughput sequencing of polymerase chain reaction (PCR)-amplified gDNA from LV tissues showed that 16.0% ± 2.9% of cardiac cells were edited at the target nucleotide (Fig. 1c), with few insertions or deletions (indels). These data underestimate editing efficiency of cardiomyocytes, which comprise only ~25% of all cardiac cells in R403Q-129SvEv mice^24^ from which gDNA was extracted. Therefore, we also assessed editing efficiency of *Myh6* transcripts (exclusively expressed in cardiomyocytes) within each cardiac chamber using RT–PCR amplification followed by high-throughput sequencing (Fig. 1d). We observed the highest editing efficiency in LV RNAs (81% ± 8%), with restoration of the normal R403 residue in cardiomyocytes. There was no significant difference between the mean editing efficiency measured in LV and RV tissues (61% ± 19%; *P* = 0.06), but significantly less editing occurred in LA (26% ± 11%) and RA (34% ± 19%) tissues compared to LV tissues (*P* = 5.5 × 10^−6^).

**Fig. 2:**
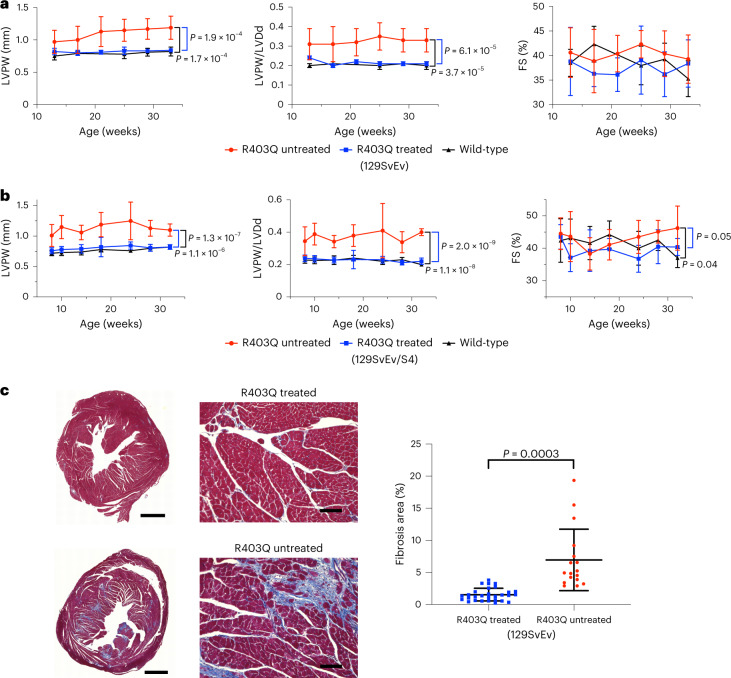
A single injection of dual-AAV9 ABE8e prevents hypertrophy and fibrosis in two strains of HCM mice. **a**,**b**, Echocardiographic measurements of the LVPW thickness (left panels), ratio of LVPW and LVDd (middle panels) and %FS (right panels) in studied mice. Untreated R403Q-129SvEv and R403Q-129SvEv/S4 developed hypertrophy (increased LVPW thickness, reduced LV volumes and increased LVPW/LVDd ratios). R403Q-129SvEv/S4 has earlier onset of hypertrophy and hypercontractility (increased FS). **a**, Longitudinal echocardiographic measurements in WT 129SvEv (*n* = 4 males; black line), untreated R403Q-129SvEv (*n* = 10 males, red line) and R403Q-129SvEv (*n* = 6 males, blue line) mice treated with a single dose of dual-AAV9 ABE8e. **b**, Longitudinal echocardiographic measurements in WT R403Q-129SvEv/S4 (*n* = 5 males, 6 females; black line), untreated R403Q-129SvEv/S4 (*n* = 6 males, 6 females; red line) and R403Q-129SvEv/S4 (*n* = 3 males, 7 females; blue line) treated with a single dose of dual-AAV9 ABE8e. **c**, Left: representative Masson-trichrome-stained LV and RV histological sections from a single dose of dual-AAV9 ABE8e-treated (upper panel) and untreated (lower panel) male R403Q-129SvEv mice. Collagen deposition (blue staining) within regions of myocardial fibrosis was prominent in the untreated mouse, whereas the histology of the treated mouse was similar to WT hearts (Fig. 3b). Scale bars, 1 mm (low magnification) and 50 μm (high magnification). Right panel: quantification of fibrosis from Masson trichrome staining in 29 ventricular sections derived from six treated male mice and 17 sections from four untreated male mice. The fibrotic load in untreated mice ranged from 2.9% to 19.4% (Extended Data Fig. 3). The untreated section shown here has a mean fibrotic load of 3.4%. Data are presented as mean values ± s.d. Significance was assessed by two-tailed *t*-test (Supplementary Information). Source data

We assessed cardiac structure and function of treated R403Q-129SvEv mice by in vivo serial echocardiography, performed every 2–4 weeks from 14 weeks of age until termination (Fig. 2a). LVH, determined by measuring the thickness of the LV posterior wall (LVPW) and ventricular remodeling, assessed by ratio of LVPW to LV end-diastolic dimension (LVDd, an index of LV geometry and reduced in HCM^4^), was increased in all untreated but not in treated R403Q-129SvEv mice, which were indistinguishable from WT mice. Fractional shortening (FS), a measure of contractility that can increase in HCM^1,2^, was similar in treated and untreated R403Q and WT mice.

R403Q-129SvEv/S4 mice were also treated with a single dose of dual-AAV9s (1.25 × 10^13^ vg kg^−1^ of each vector) carrying ABE8e (Fig. 1b). Serial in vivo echocardiography performed every 2–4 weeks from 8 weeks of age showed LVH in all untreated female and male R403Q-129SvEv/S4 mice at 10 weeks with subsequent ventricular remodeling and hypercontractility (Fig. 2b). By contrast, cardiac morphology and function of treated R403Q-129SvEv/S4 mice did not show either rapid or insidious onset of any of these HCM phenotypes. Instead, treated mice remained indistinguishable from WT mice throughout the 32 weeks of study duration.

Together, these data indicated that base editing of the mutant R403Q allele, delivered several months or only weeks in advance of the typical time for disease manifestations, resulted in durable prevention of ventricular remodeling and hypertrophy.

### Base editing prevents cardiac fibrosis

**Extended Data Fig. 3:**
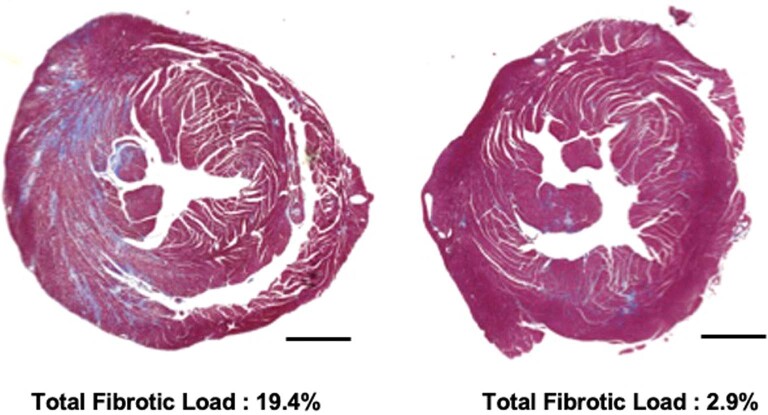
Cardiac histopathology in untreated R403Q-SvEV mice. Representative Masson-trichrome stained ventricular sections (short axis view) from two untreated R403Q-129SvEv mice. Across all sections of untreated mice the extent of collagen deposition (blue staining), indicative of regions of myocardial fibrosis, was assessed. Shown here are sections from two mice with a mean percentage of myocardial fibrosis equaling 2.9% (right) and 19.4% (left). Scale bars: 1 mm.

Myocardial fibrosis is prominently increased in mouse models of HCM^11,12,21^. In human patients, fibrosis contributes to adverse outcomes, including heart failure and arrhythmias^25,26^. We studied microscopic cardiac histology in ventricular sections of WT and treated and untreated R403Q-129SvEv mice (34 weeks of age) to determine the impact of editing on myocardial fibrosis (Fig. 2c and Extended Data Fig. 3). Similarly to previous studies, untreated R403Q-129SvEv mice revealed increased amounts of focal and diffuse fibrosis in comparison to WT mice. Treated mice had minimal fibrosis, similarly to that in WT mice, and significantly less than in untreated mice (*P* = 0.0003).

### Assessment of bystander editing and histopathology

**Extended Data Fig. 4:**
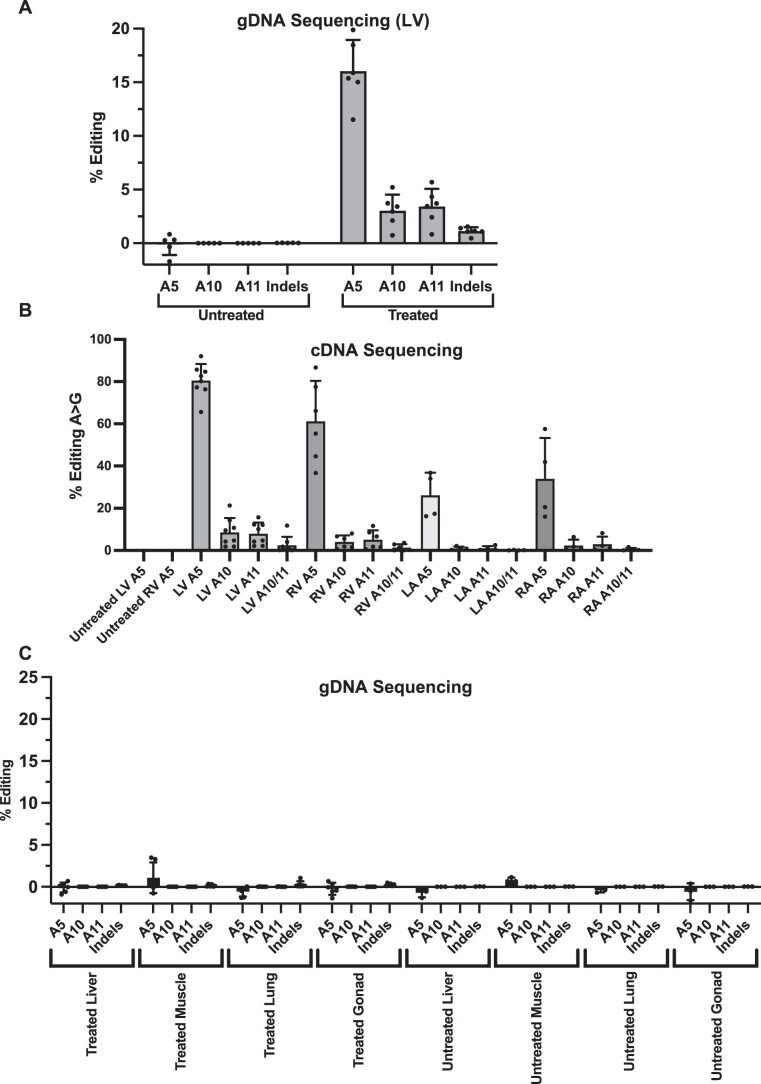
Bystander edits assessed after single injection of AAV9-ABE8e. **A)** Editing efficiencies (%) measured after a single injection of AAV9-ABE8e based on high-throughput sequencing of PCR product amplified from LV gDNA (untreated n = 5, treated n = 6), quantified by CRISPResso2 (https://github.com/pinellolab/CRISPResso2). Editing efficiency at the on-target site A5 is compared to editing at bystander nucleotides A10 and A11. Indel rates were also assessed. **B)** Editing efficiencies (%) in untreated mice and mice treated with a single dose of AAV9-ABE8e. High-throughput sequencing of PCR product amplified from *Myh6* cDNA derived from untreated LV and treated LV (n = 8), RV (n = 6), LA (n = 4) and RA (n = 4) identified bystander nucleotides A10, A11 or both (A10/11). **C)** Editing efficiency (%) assessed by sequencing PCR amplified gDNA extracted from non-targeted tissues: liver, skeletal muscle, lung and gonads from 6 treated and 3 untreated mice. Note low levels of on target *Myh6* editing of the pathogenic variant R403Q (A5) in these non-targeted tissues. Data are presented as mean values ±SD. Source data

We assessed the frequency of bystander mutations in base-edited R403Q mice, as amino acid substitutions could be deleterious. Using gDNA and RT–PCR-amplified *Myh6* mRNAs from cardiac tissues extracted from treated R403Q-129SvEv mice, we determined the editing of bystander adenosine residues at protospacer positions 10 and 11 (Fig. 1a) and the introduction of indels. On average, the most common bystander edit was 3.4% ± 1.7% in gDNA and 5.0% ± 4.8% in RNA (Extended Data Fig. 4a,b); few indels were observed (<2%). No bystander edits occurred in alleles that maintained the pathogenic R403Q. Moreover, sequence data indicated a correlation between the proportions of appropriate editing at the A5 pathogenic nucleotide and bystander editing: A10: *r* = 0.68, *P* = 2 × 10^−12^; A11: *r* = 0.45, *P* = 2 × 10^−5^.

We also compared on-target (A5) and bystander editing in gDNA from LVs, livers, skeletal muscles, lungs and gonads from treated R403Q-129SvEv mice (Extended Data Fig. 4c). Very low levels of on-target editing were observed, and virtually no bystander editing occurred in extra-cardiac tissues, consistent with the *Tnnt2* promoter’s cardiomyocyte-specific expression.

**Extended Data Fig. 5:**
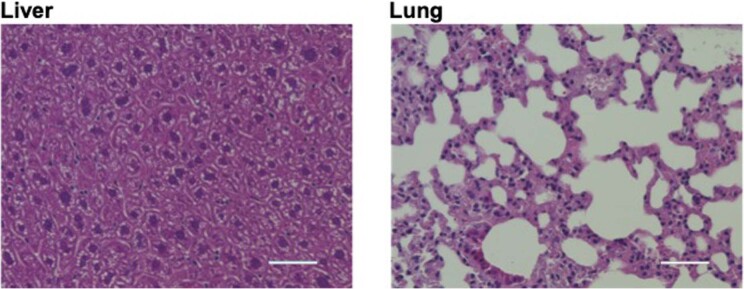
Liver and lung histology after AAV9-ABE8e injection. Representative sections of liver (left) and lung (right) obtained from R403Q-129SvEv mice (n = 3) injected with 1.1×10^13^ AAV9-ABE8e vg/kg showed normal architecture and no infiltrates of inflammatory cells.

Recognizing the potential for cellular damage to the lungs after intra-thoracic injections and the liver due to tropism by AAV9 (ref. ^27^), we studied the histology of these organs in treated R403Q-129SvEv mice. Lung and liver cell architecture remained normal and showed no enrichment of inflammatory cells. Serologic assessment of liver enzymes, bilirubin and globulins were also normal (Extended Data Fig. 5 and Supplementary Table 2).

### Assessment of two doses of base editor AAV9

Although a single injection of dual-AAV9 carrying ABE8e prevented the development of HCM in all treated R403Q mice, the editing efficiency varied among mice and was lower in RV and atrial tissues than in LV (Fig. 1d). As HCM can pathologically remodel the RV^28^ and atria^29^, we tested whether a second dose of the dual-AAVs could improve editing in these chambers. Because immune responses are not elicited in mice when two doses of AAV9 are delivered in close proximity and before neonatal day 14 (ref. ^30^), we delivered a second dose within 1 week of the first dose to R403Q-129SvEv/S4 mice (Fig. 1b).

**Fig. 3:**
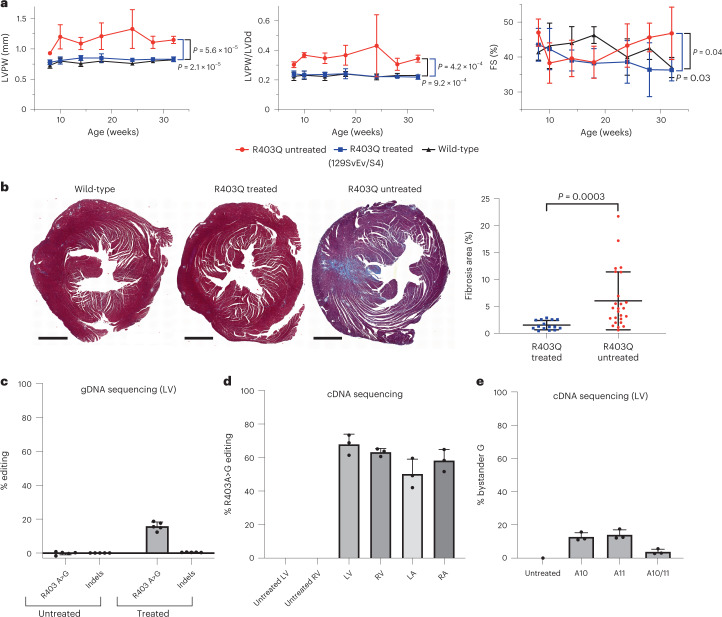
Two doses of dual-AAV9 ABE8e increase atrial but not ventricular base editing. **a**, Longitudinal echocardiographic measurements (defined in Fig. 2) of the LVPW (left panel), LVPW/LVDd ratio (middle panel) and %FS (right panel) in WT 129SvEv/S4 (*n* = 5 males, black line), untreated R403Q-129SvEv/S4 (*n* = 6 males, red line) and R403Q-129SvEv/S4 mice treated with two doses of dual-AAV9 ABE8e (*n* = 6 males, blue line). Untreated but not treated R403Q-129SvEv/S4 developed early-onset hypertrophy (increased LVPW thickness and increased LVPW/LVDd) and hypercontractility (increased FS). **b**, Left: representative Masson-trichrome-stained LV and RV histological sections from WT 129SvEv/S4 male (left), treated (two doses of AAV9 ABE8e) male R403Q-129SvEv/S4 (middle) and untreated R403Q-129SvEv/S4 (right) male mice. Collagen deposition (blue staining) within regions of myocardial fibrosis was prominent in the untreated but minimal in the treated mouse and WT mice. (Scale bar, 1 mm). Right panel: quantification of fibrosis from Masson-trichrome-stained ventricular sections (five per mouse) from untreated (*n* = 5 males) and treated (*n* = 3 males; two doses) of AAV9 ABE8e R403Q-129SvEv/S4 mice. **c**, Editing efficiency of the targeted R403Q allele and indels was assessed in LV gDNA derived from five treated and five untreated male mice. **d**, Editing efficiency was assessed by sequencing *Myh6* cDNA derived from RNAs extracted from the LVs, RVs, LAs and RAs from three male mice. Atrial editing was increased after two doses. **e**, The mean percent of bystander edits was detected in pooled LV cDNAs from R403Q-129SvEv/S4 mice (*n* = 3 males) treated with two doses. Data are presented as mean values ± s.d., and significance was assessed by two-tailed *t*-test (Supplementary Information). Source data

Mice treated with two doses had similar feeding and activity levels to untreated and single-dose-treated R403Q-129SvEv/S4 mice, and all survived throughout the 32 weeks of study. Serial echocardiograms (Fig. 3a) showed no emergence of LVH, ventricular remodeling or hypercontractility in mice treated with two doses compared to untreated R403Q-129SvEv/S4 mice. There was marked attenuation of myocardial fibrosis (*P* = 0.0003; Fig. 3b). Serologic assessment of liver enzymes, bilirubin and globulins from mice treated with two doses was normal (Supplementary Table 2).

**Extended Data Fig. 6:**
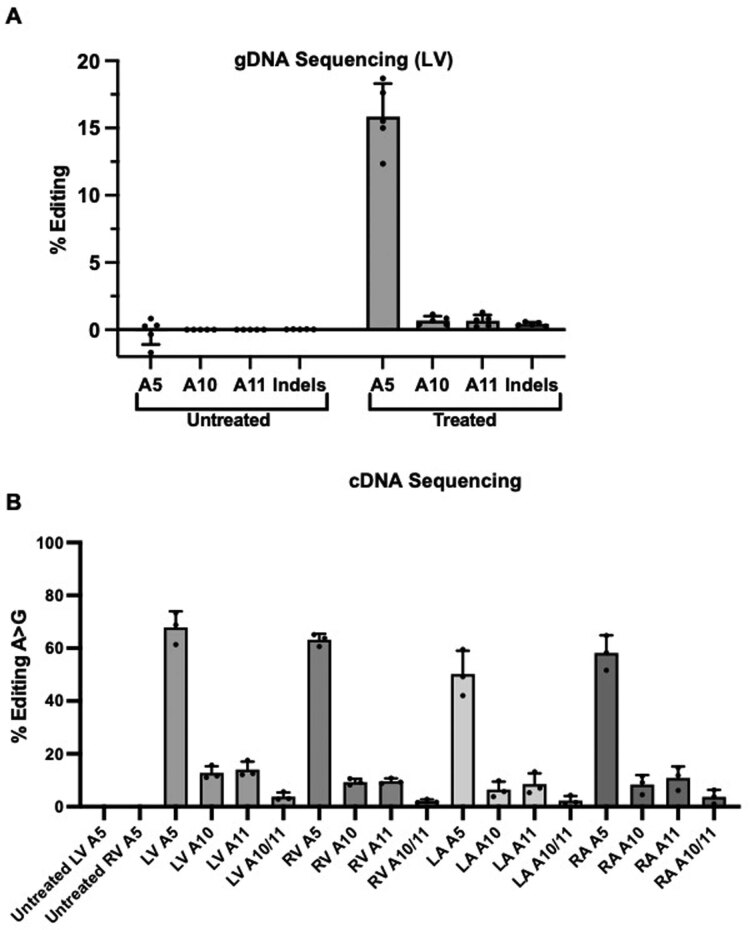
Bystander editing analysis after two injections of AAV9-ABE8e in each cardiac chamber. **A)** Editing efficiency (%) of the targeted pathogenic variant R403Q based on high-throughput sequencing of PCR product amplified from gDNA extracted from LV tissues from untreated (n = 5) and treated (n = 5; two doses of AAV9-ABE8e) mice. Editing efficiency at the dominant heterozygous R403Q pathogenic variant is calculated as: (wild type nucleotide percentage – 50%) divided by 50%. **B)** Editing efficiencies (%) after two injections based on high-throughput sequencing of PCR products amplified from *Myh6* cDNA derived from RNA extracted from the LV (n = 3), RV (n = 3), LA (n = 3), and RA (n = 3) at bystander nucleotides A10, A11 or both (A10/11). Data are presented as mean values ±SD. These data are summarized in Fig. 3. Source data

Unexpectedly, the additional dose did not significantly increase the mean editing efficiency in LV and RV (Fig. 3c,d). Analyses of LV gDNA showed 15.8% ± 2.5% editing with two doses (Fig. 3c and Extended Data Fig. 6a) compared with 16.0% ± 2.9% editing with one dose (Fig. 1c and Extended Data Fig. 4a). RNA analyses showed similar editing efficiencies after two and one doses in LV (68% ± 6% versus 81% ± 8%) and RV (63% ± 2% versus 61% ± 19%). By contrast, the editing efficiencies in atria improved with two doses compared to one dose (LA: 50% ± 9% versus 26% ± 11%, *P* = 0.02; RA: 58% ± 7% versus 34% ± 19%, *P* = 0.08) (Figs. 1d and 3d).

Sequence analyses of gDNA and cDNAs from cardiac tissues of mice treated with two doses identified bystander edits (Fig. 3e and Extended Data Fig. 6a,b). In comparison to the bystander edits after one dose (Extended Data Fig. 4b; *n* = 24 tissues), cDNA analyses of mice treated with two doses (Extended Data Fig. 6b; *n* = 14 tissues) indicated increased bystander editing at A10 (9.2% ± 3.3% versus 4.8% ± 5.3%, *P* = 0.006) and A11 (10.8% ± 3.6% versus 5.0% ± 4.8%, *P* = 0.0005).

### R403Q correction normalizes the transcriptome

**Extended Data Fig. 7:**
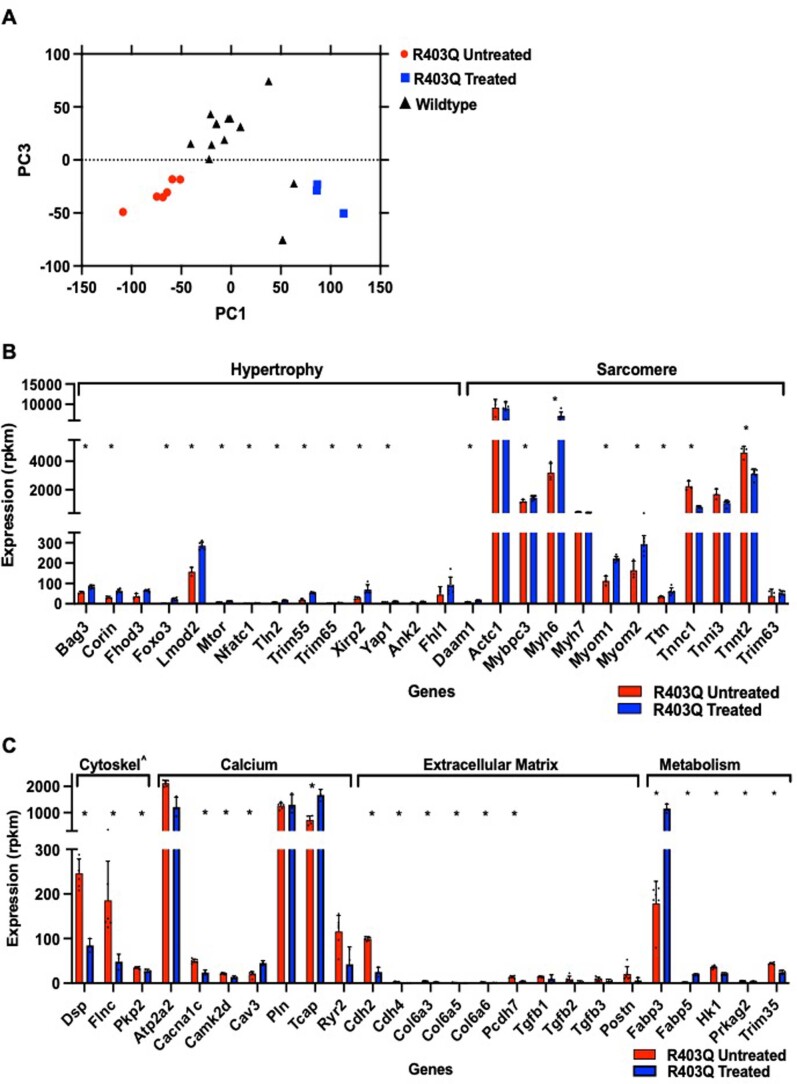
LV RNA expression in WT, treated R403Q and untreated R403Q mice. **A)** Principal component analyses of WT (n = 12, black triangles), R403Q-129SvEv/S4 (n = 6, red circles) and treated (2 doses of AAV9-ABE8e) R403Q-129SvEv/S4 (n = 3, blue squares) mice. Principal component 1 (PC1) provided the best discrimination of phenotype and treatment, accounting for 25% of the variance. PC3 accounted for 12% of the variance. **B)** and **C)** Expression levels of genes involved in development of LV hypertrophy, sarcomere structure, cytoskeletal function, calcium handling, extracellular matrix and metabolism were assessed in untreated (n = 6) and treated (two doses of AAV9-ABE8e; n = 3) R403Q-129SvEv/S4 mice. * denotes RNAs with significantly (p < 0.05) altered expression in treated compared to untreated R403Q-129SvEv/S4. Data are presented as mean values ±SD and significance assessed by two-sided T-test (provided in Supplementary Table S3). Source data

We assessed transcriptional responses by RNA sequencing (RNA-seq) analyses (Methods) of LV tissues from WT, untreated and treated R403Q-129SvEv/S4 mice (Extended Data Fig. 7 and Supplementary Table 3). Principal component analyses comparing all expressed transcripts (*n* = 12,035 RNAs with ≥3 RPKM expression in any group) showed greater similarities between WT and treated compared to untreated R403Q-129SvEv/S4 mice. Principal components 1 and 3 explained 25% and 12% of the variance, respectively, between WT and R403Q-129SvEv/S4 (Extended Data Fig. 7a). We then selectively compared transcripts with recognized involvement in hypertrophic remodeling in mouse models and human patients with HCM^12,21,31^. Although gene expression varied among mice within the same treatment group, overall the mean fold changes in transcript levels (Supplementary Table 3) were consistent with greater mechanical work, metabolic stress and extracellular matrix expansion in untreated compared to treated mice. Untreated mice expressed higher levels of transcripts encoding contractile proteins (*Myh7*, *Mybpc3*, *Myom1*, *Myom2* and *Ttn*), associated with hypertrophic growth (*Ank2*, *Daam1*, *Fhl*, *Trim 63* and *Yap*) and enabling a metabolic shift from normal fatty acid utilization (*Fabp*, higher in treated mice) toward glycolysis (*mTOR*, *Prkag2* and *HK1*). Consistent with histopathologic findings (Figs. 2c and 3b), the expression of fibrogenic genes (*Col6a5*, *Col6a6*, *Postn*, *Tgfb2* and *Ctgf*) were higher in untreated versus treated mice.

### Assessment of genome-wide off-target editing

**Extended Data Fig. 8:**
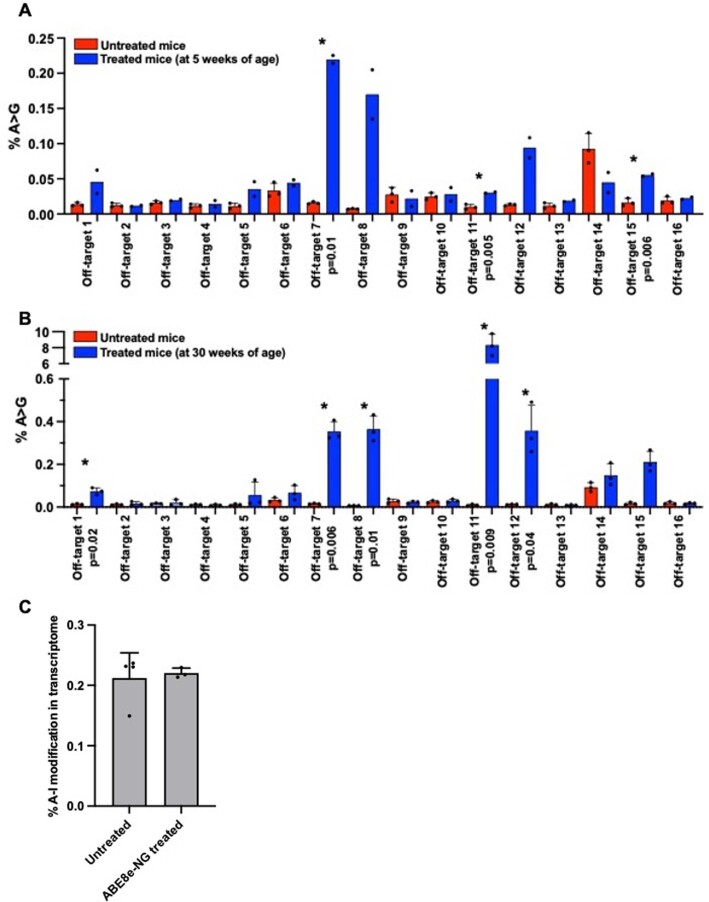
Off-target editing analysis in AAV9-ABE8e treated mice. Candidate genomic loci identified by CIRCLE-seq (see Methods) were PCR-amplified and sequenced by high throughput DNA sequencing (Illumina MiSeq). Adenine base editing at the nucleotide closest to the window center was quantified by CRISPResso2 (https://github.com/pinellolab/CRISPResso2). **A)** Detected Off-target editing in untreated mice (n = 3) compared to mice receiving two doses of base editor AAVs sacrificed at 5 weeks of age (n = 2). **B)** Detected off-target editing in untreated mice (n = 3) compared to mice receiving two doses of base editor AAVs sacrificed at 30 weeks of age (n = 3). **C)** Assessment of RNA editing (A-to-I nucleotide changes) by ABE8e in RNA sequencing data from LV tissues of treated (n = 3) and untreated (n = 4) R403Q mice. Data are presented as mean values ±SD and significance assessed by two-sided T-test (provided in Source Data). Source data

Distinct from bystander editing (in proximity to the target nucleotide), off-target editing occurs when ABE8e binds distinct genomic loci with homology to the guide sequence. We used CIRCLE-seq to identify potential genome-wide off-target loci (Extended Data Fig. 8 and Supplementary Tables 4 and 5) that could be bound by the sgRNA-SpCas9-NG complex^32^. After PCR amplification of potential off-target editing loci from genomic LV DNA, we performed next-generation sequence analyses. Many of the top identified loci fell in repetitive sequences that we could not specifically PCR amplify to assess editing, but we suggest that off-target editing within such repetitive sequences would be unlikely to be deleterious. Out of 16 successfully amplified potential off-target loci, low (<0.25%) but statistically significant editing occurred at three sites in 5-week-old treated mice (Extended Data Fig. 8a and Supplementary Table 4). Analyses of treated and untreated 30-week-old mice (Extended Data Fig. 8b and Supplementary Table 4) revealed 8.3% ± 1.1% A>G editing at off-target site 11, an intergenic chromosome 3 region that is 26 kb from the nearest predicted gene (*Gm26107*) with unknown functions. Four other tested loci residing at intergenic or intronic regions had low but statistically significant off-target editing (<0.4% at each site).

### Assessment of editing across the transcriptome

Cellular RNA is a known substrate of ABE8e, and off-target RNA editing was reported after lipofection of ABE8e into HEK293T cells^20^. We assessed off-target RNA editing by quantifying A-to-I nucleotide changes in LV RNA-seq data obtained from treated and untreated R403Q mice. Similarly to previous analyses of animals treated with AAV vectors encoding constitutively expressed ABEs^33,34^, no significant differences were observed in the frequency of A-to-I RNA nucleotide changes in treated and untreated mice (Extended Data Fig. 8c).

### Eliminating mutant alleles with Cas9 nuclease to prevent HCM

As RNAi selectively silences R403Q transcripts in mice and prevents HCM development for approximately 6 months without adverse consequences^14^, we explored the effects of base editors to permanently inactivate the mutant allele. This strategy could overcome the need to design base editors to specifically correct each pathogenic variant by editing common, benign polymorphisms to yield allele-specific, frameshift inactivation. Moreover, as human genomic data indicate that loss of functional variants in one myosin allele are tolerated (*MYH7* pLI = 0), half of the normal myosin gene dosage appears adequate for proper cardiac function in mice^35^ and humans.

**Fig. 4:**
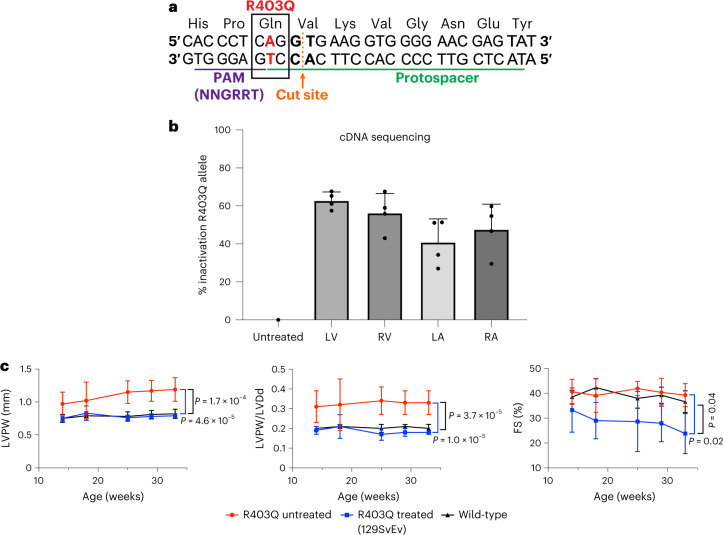
AAV9-Cas9 silencing of the pathogenic variant R403Q and cardiac function in R403Q-129SvEv mice. **a**, Schematic of the genomic sequence surrounding the HCM R403Q pathogenic variant, showing the pathogenic variant R403Q (numbered according to the human *MYH7* amino acid residues) and flanking amino acid residues (N, amino terminus; C, carboxyl terminus). The *S. aureus* Cas9 nuclease protospacer (green line), PAM site (purple line) and double-stranded cleavage position of the nuclease (dotted orange line) are shown. **b**, The percent of inactivation of the R403Q allele after 1 × 10^13^ vg kg^−1^ of AAV9-Tnnt2-*S. aureus*-Cas9 (designated AAV9-Cas9) was assessed by sequencing *Myh6* cDNA, amplified from RNA extracted from each cardiac chamber of surviving R403Q-129SvEv mice (*n* = 4 males) at 33 weeks. The percentage of inactivation of the pathogenic variant R403Q was calculated as: (1 − (total number of R403Q reads, divided by total number of WT reads)) multiplied by 100. **c**, Longitudinal echocardiographic measurements (defined in Fig. 2) of the LVPW (left panel), LVPW/LVDd ratio (middle panel) and %FS (right panels) in WT R403Q-129SvEv (*n* = 4 males; black line), untreated R403Q-129SvEv (*n* = 10 males, red line) and treated (AAV9-Cas9) R403Q-129SvEv (*n* = 5 males; blue line) mice. Note impaired contractile function (FS <40%) in treated mice. Data are presented as mean values ± s.d., and significance was assessed by two-tailed *t*-test (Supplementary Information). Source data

We aimed to selectively inactivate the R403Q allele in cardiomyocytes, by introducing indels with *S. aureus* Cas9 nuclease expressed via the *Tnnt2* promoter. Unlike ABEs, the therapeutic cargo of *S. aureus* Cas9 nuclease and sgRNA can be packaged into a single AAV9 vector (Extended Data Fig. 2). We designed the sgRNA for the *S. aureus* Cas9 nuclease so that the target R403Q is within the spacer region, directly adjacent to the PAM site (Fig. 4a). After nuclease cleavage of DNA, non-homologous end-joining events will inactivate mutant alleles. Although this design specifically targeted the R403Q variant, similar approaches could effectively target common heterozygous variants that are specific to the myosin gene copy with any HCM pathogenic variant.

After intra-thoracic injection delivery of AAV9-Cas9 nuclease (1.1 × 10^13^ vg kg^−1^) to R403Q-129SvEv mice (*n* = 5) (Fig. 1b), we assessed inactivation of the R403Q allele using RT–PCR amplification of LV RNA and high-throughput sequence analyses. Few transcripts contained frameshifts, presumably because most mRNAs transcribed from indel alleles underwent nonsense-mediated decay. As such, we calculate the percent of R403Q allele inactivation as (1 – (total number of R403Q reads, divided by total number of WT reads)) multiplied by 100.

AAV9-Cas9 nuclease efficiently inactivated the R403Q in cardiac ventricles (LV: 63% ± 5%; RV: 56% ± 10%) but less so in atria (LA: 41% ± 12%; RA: 48% ± 13%; ventricular versus atria, inactivation: *P* = 0.01; Fig. 4b). Echocardiography of treated mice showed no development of LVH or ventricular remodeling, unlike untreated R403Q-129SvEv mice (Fig. 4c, panels LVPW and LVPW/LVDd). However, the contractile function (LV FS) of treated mice decreased by 12 weeks of age and remained significantly reduced throughout the 33 weeks of study (Fig. 4c).

**Extended Data Fig. 9:**
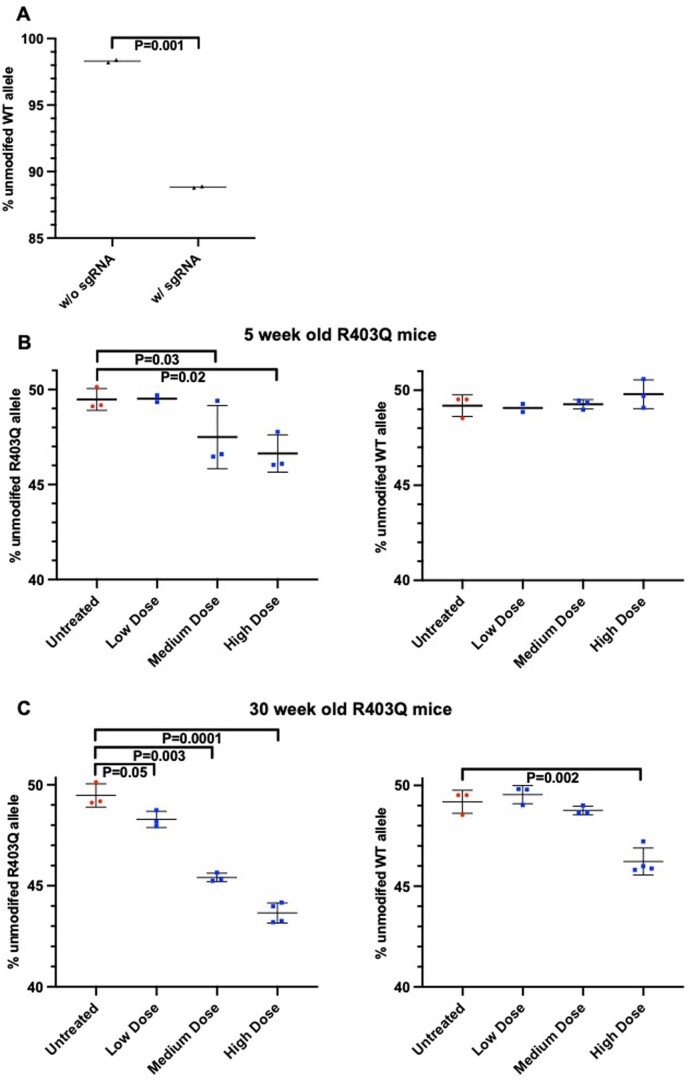
Genomic DNA sequencing of mice treated with AAV9-Cas9 nuclease. **A)** The frequency of unmodified WT alleles measured from high-throughput sequencing of PCR products amplified from the targeted locus in gDNA from the LV of WT mice treated with high dose (1.1×10^13^) AAV9-Cas9 with sgRNA (n = 2) or without sgRNA (n = 2). **B)** and **C**) The frequency of unmodified WT and R403Q alleles measured from high-throughput sequencing of the targeted genomic locus in gDNA from LVs of 3-4 untreated mice and mice treated with low, medium, and high doses of AAV9-Cas9 mice after 5 weeks **(B)** and 30 weeks **(C)**. Data are presented as mean values ±SD and significance was assessed by two-sided T-test. Source data

We assessed whether the deleterious consequences of Cas9 treatment reflected non-specific toxicity caused by Cas9 protein or unintended targeting of the WT allele by treating WT mice for 30 weeks with 1.1 × 10^13^ vg kg^−1^ of AAV9-Cas9 nuclease with (*n* = 2) and without (*n* = 2) sgRNA. All WT mice remained healthy and had normal cardiac function (FS: 40% ± 4% with guide; 39% ± 8% without guide). However, high-throughput sequencing revealed that mice receiving AAV9-Cas9 nuclease and sgRNA had disruption of ~9% of the WT allele despite the one nucleotide mismatch to the sgRNA (Extended Data Fig. 9a; *P* = 0.001).

From these data, we deduced that cardiac dysfunction in heterozygous R403Q mice receiving high doses of AAV9-Cas9 nuclease with targeting sgRNA likely reflected unintended disruption of the WT allele. This event could result in hemizygous R403Q cardiomyocytes with profoundly depressed contractile performance—similar to mice with homozygous R403Q alleles that die as neonates^36^. Alternatively, concurrent disruption of the WT allele and the introduction of an indel in the R403Q allele could ablate *Myh6* expression and cause cardiomyocyte lethality.

**Fig. 5:**
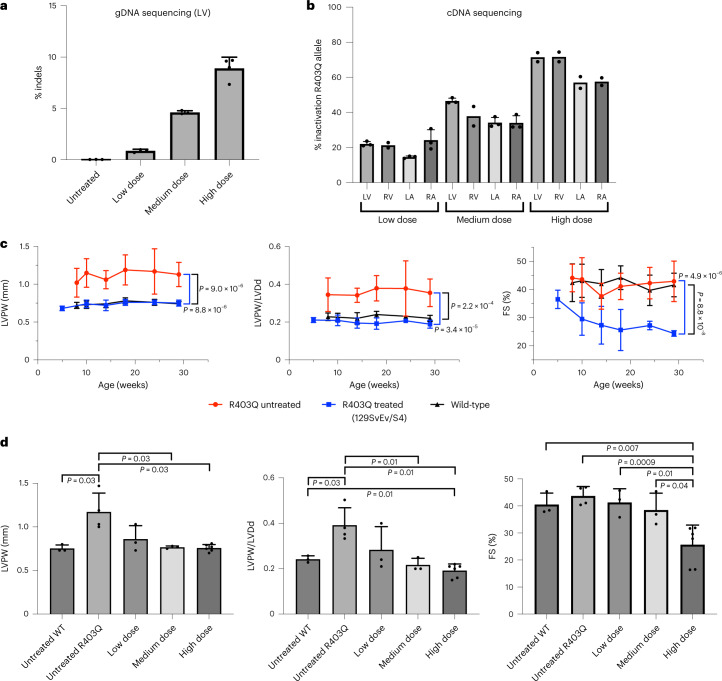
Assessment of R403Q-allele inactivation and echocardiographic findings in R403Q-129SvEv/S4 mice treated with low, medium and high doses of AAV9-Cas9. **a**, Percent of R403Q alleles with indels in three untreated male or AAV9-Cas9 treated (low dose: *n* = 1 male, 2 females; medium dose, *n* = 3 females and high dose, *n* = 4 females) R403Q-129SvEv/S4 mice was assessed by next-generation sequencing of PCR-amplified LV gDNA and analyzed using CRISPResso2. **b**, *Myh6* cDNAs, amplified from RNAs extracted from each cardiac chamber of R403Q-129SvEv/S4 mice, treated with low (1.1 × 10^12^ vg kg^−1^; *n* = 1 male, 2 females), medium (5.4 × 10^12^ vg kg^−1^; *n* = 3 females) and high (2.2 × 10^13^ vg kg^−1^; *n* = 2 females) doses of AAV9-Cas9 for 18–33 weeks. The percentage of inactivation of the pathogenic variant R403Q was calculated as: (1 – (total number of R403Q reads, divided by total number of WT reads)) multiplied by 100. **c**, Longitudinal echocardiographic measurements LVPW, LVPW/LVDd and FS (defined in Fig. 2) in WT 129SvEv/S4 (*n* = 5 males, 5 females, black line) and untreated R403Q-129SvEv/S4 (*n* = 5 males, 6 females, red line) and treated (AAV9-Cas9 2.2 × 10^13^ vg kg^−1^) R403Q-129SvEv/S4 (*n* = 6 females, blue line). Note impaired contractile function (FS <40%) in high-dose-treated mice. **d**, Echocardiographic measurements of LVPW thickness, LVPW/LVDd ratio and FS (defined in Fig. 2) in R403Q-129SvEv/S4 mice at 20 weeks of age treated with low (1.1 × 10^12^ vg kg^−1^, *n* = 1 male, 2 females), medium (5.4 × 10^12^ vg kg^−1^, *n* = 3 females) and high (2.2 × 10^13^ vg kg^−1^, *n* = 6 females) doses of AAV9-Cas9, compared to untreated WT 129SvEv/S4 (*n* = 3 females) and untreated R403Q-129SvEv/S4 (*n* = 4 females) mice. Note that one mouse treated with the low dose had increased LVPW and increased LVPW/LVDd, indicating a non-therapeutic response. Data are presented as mean values ± s.d., and significance was assessed by two-tailed *t*-test (Supplementary Information). Source data

We then studied the effects of three doses—low (1.1 × 10^12^ vg kg^−1^), medium (5.4 × 10^12^ vg kg^−1^) and high (1.1–2.2 × 10^13^ vg kg^−1^)—of AAV9-Cas9 with sgRNA delivered to R403Q-129SvEv/S4 mice. High-throughput sequencing of the target site in LV gDNA revealed a dose-dependent increase in the percentage of indels (Fig. 5a). We observed a strong correlation between viral dose and the percent of the R403Q allele inactivation from high-throughput sequencing of cDNA (*P* = 1 × 10^−6^, *r* = 0.69; Fig. 5b). Inactivation of the R403Q allele was significantly higher in ventricles (mean inactivation: 72% ± 3%) than atria (mean inactivation: 57% ± 3%, *P* = 0.0007; Fig. 5b).

**Extended Data Fig. 10:**
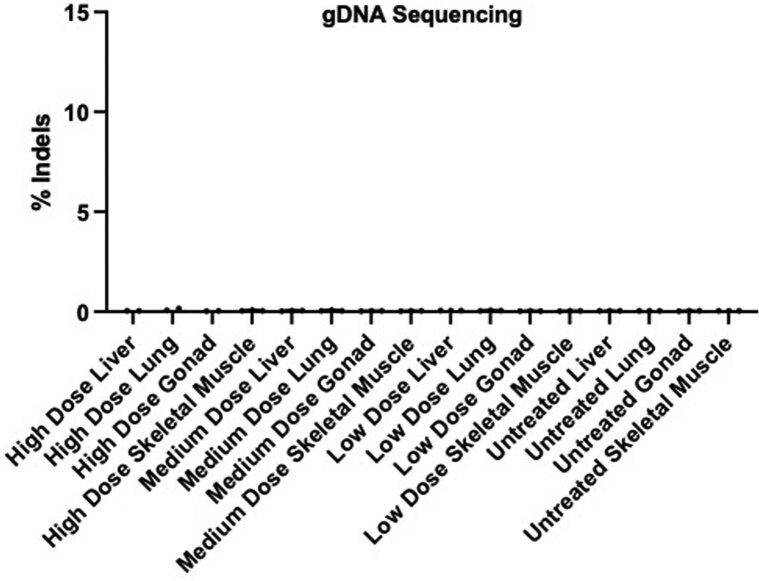
Assessment of Indels from extracardiac tissues derived from mice treated with AAV9-Cas9 nuclease. Indels were measured by high-throughput sequencing of PCR products amplified from the targeted *Myh6* region in gDNA extracted from the liver, lung, gonad and skeletal muscle (quadricep) of mice treated with AAV9-Cas9 nuclease (n = 2-3 per dose) and from untreated mice (n = 3). Reads were aligned to the target site by CRISPResso2. CRISPResso2 quantification of indels is plotted for each sample. Source data

Analyses of LV gDNA also indicated that Cas9 nuclease caused significant and dose-dependent loss of the WT allele over time (Extended Data Fig. 9b,c). Tissues harvested at 5 weeks showed no loss of the WT allele in mice treated with any tested dose, although loss of the R403Q allele was evident with the medium or high dose. Analyses of treated mice at 30 weeks showed that high dose of AAV9-Cas9 caused loss ~4% of the WT allele, corresponding to allelic loss in ~8% of cells. Because cardiomyocytes comprise approximately 25% of cardiac cells and nuclease expression was restricted to cardiomyocytes, we estimate that 32% of cardiomyocytes carried a disrupted WT *Myh6* allele in mice treated with the high dose of AAV9-Cas9. Disruption of *Myh6* in the liver, lung, gonad and skeletal muscle of animals treated with high doses of AAV9-Cas9 was not observed (Extended Data Fig. 10).

Serial echocardiography also showed dose-dependent responses to Cas9 nuclease treatment. High-dose treatment prevented the emergence of LVH (Fig. 5c,d) but consistently depressed contractile function (Fig. 5d). Two mice with depressed FS and enlarged LV developed heart failure (fluid retention, low food intake and lethargy), necessitating sacrifice at 21 weeks. Although the remaining mice treated with a high dose of AAV9-Cas9 survived throughout the 33 weeks of study, none recovered normal cardiac contractility.

In contrast, mice treated with medium and low doses survived and had similar vitality to WT mice. There were also no significant differences in mean contractility or mean ventricular dimensions among mice treated with medium or low dose nor between either medium or low dose and WT mice (Fig. 5D). LV wall thickness remained normal in all mice treated with the medium dose; however, one mouse treated with the low dose developed LVH and reduced LV volumes (Fig. 5d; highest LVPW/LVDd value). Analyses showed similar inactivation of the R403Q allele among all low-dose-treated mice (Extended Data Fig. 9), thereby excluding technical issues with AAV administration.

From these data, we conclude that low doses of Cas9 nuclease were inadequate to consistently inactivate the R403Q allele in mice, whereas high doses corrected hypertrophy but led to depressed contractile shortening. The medium dose appears to comprise a suitable intermediate that consistently corrected hypertrophy and did not result in heart dysfunction.

## Discussion

We demonstrate efficient and durable in vivo base editing of a pathogenic single-nucleotide variant in myosin that is abundantly and selectively expressed in cardiomyocytes. Correction of ~70% of ventricular R403Q transcripts was sufficient to prevent the morphologic, histopathologic and molecular hallmarks of HCM in mice. Further development and translation of this approach to correct the most serious human pathogenic variants would have considerable clinical impact and the potential to cure HCM and other genetic cardiomyopathies.

Similarly to our previous studies that employed RNAi to selectively silence the R403Q allele, we found that permanent genomic correction of all cardiomyocytes was unnecessary to prevent the emergence of HCM. snRNA-seq showed that ~55% of LV cardiomyocytes expressed ABEs, providing a lower-bound estimate due to limited sequencing read depth, whereas targeted sequencing of *Myh6* transcripts showed editing in over 70% of R403Q alleles. As there were no overt functional consequences in mice with variable amounts of editing within or between cardiac chambers, tissue mosaicism comprising corrected and uncorrected cardiomyocytes was not deleterious. Editing of atria was less efficient compared to ventricles, perhaps reflecting lower atrial coronary blood flow and perfusion pressures^37,38^ that limited vector delivery. An additional dose improved atrial editing, which could prevent atrial remodeling that increases atrial fibrillation and thromboembolic events in patients with HCM^4^. Longitudinal studies are needed in larger animals to discern if gene editing or silencing influences cardiac hemodynamics and arrhythmic susceptibility.

We observed modest bystander editing only on the R403Q allele, indicating preferential editing of the target nucleotide. Bystander edits could substitute the normal lysine (position 405) with an arginine (no change in charge), glutamic acid or glycine (both change the charge). These substitutions are absent from population (gnomAD^39^) and disease (ClinVar;^40^ Cardiomyopathy^3^) databases. We also observed very low off-target editing at 5 of 16 potential loci, but, at longer durations of exposure to the editor, one site reached ~8% editing efficiency. Off-target loci were within mouse intronic or intergenic genomic regions. Although neither bystander nor off-target editing caused adverse outcomes in treated mice, further studies in human cells and non-human primates are needed to define long-term risk. Pre-clinical studies might also explore weaker cardiac promoters and ABEs with reduced off-target editing, such as ABE8e-V106W^41^, ABE7.10 (ref. ^42^) and ABE8.17 or ABE8.20 (ref. ^43^).

An important obstacle to treating all patients with HCM is the considerable diversity of pathogenic variants (including over 250 myosin missense residues), which makes clinical development of single-variant correction approaches difficult. We demonstrate that gene disruption by nucleases can address multiple dominant variants, such as R403Q, in genes that tolerate haploinsufficiency. Although this approach successfully prevented LVH in R403Q mice, high doses of AAV-Cas9 nuclease also disrupted the WT allele and likely accounted for impaired cardiac contractility. Using higher-fidelity Cas nuclease variants or prime editors^44,45^ may enhance allelic specificity and pose lower risks for inactivating the WT allele.

The phenotypic improvements in edited mice with rapid and insidious onset of HCM indicated a broad clinical window of opportunity to correct the consequences of pathogenic variants. The delivery of editors within weeks of the predicted time for LVH appearance in R403Q-129SvEv/S4 mice was as effective in preventing HCM as treating R403Q-129SvEv mice many months before disease develops. In addition to preventing morphologic and histopathological abnormalities, transcriptional signatures of gene programs, indicating cardiomyocyte hypertrophy, metabolic stress and activation of fibrogenesis, were normalized in both HCM models.

We expect that the temporal proximity of an effective preventive intervention and disease onset will be an advantageous feature for clinical translation of these approaches. Recent clinical studies have defined biomarkers that identify carriers of pathogenic variants with imminent onset of HCM^46^. We expect that these biomarkers will appropriately optimize the timing for productive genetic interventions while withholding these interventions from carriers of pathogenic variants with additional years of good health.

In conclusion, we established therapeutic windows, risks and opportunities in a mammalian model for base editor and Cas9 nuclease interventions to prevent HCM. These can form the basis of one-time cures for cardiomyopathies.

## Methods

Mouse experiments were reviewed and approved by the Harvard Medical School Animal Care and Use Committee and complied with all relevant ethical regulations. No data points were excluded from analyses. Source data for all experiments are provided as individual .xlsx files.

### Mouse husbandry and AAV9 administration

Mice strains used in these studies included 129S6/SvEvTac from Taconic Biosciences (https://www.taconic.com) and 29S4/SvJaeJ (009104) from Jackson Laboratory (https://www.jax.org). All mice were maintained in a virus-free animal facility with 12-hour dark/light cycles and ambient temperature and humidity.

A mouse model of HCM was previously constructed^11^ by introducing a heterozygous missense residue R403Q into the α-cardiac myosin heavy chain gene (*Myh6*) of 129SvEv mice (designated as R403Q-129SvEv). As only male R403Q-129SvEv mice consistently develop LVH and myocardial fibrosis by 20–25 weeks, treatment effects on cardiac morphology were studied only in male mice. Treated female R403Q-129SvEv mice were studied to assess editing efficiencies. Both male and female F1 hybrid R403Q-129SvEv and 129S4/SvJaeJ mice (designated R403Q-129SvEv/S4) exhibit HCM phenotypes of LVH and myocardial fibrosis by 8–10 weeks, and both sexes were studied to assess treatment effects of editing or silencing efficiency.

Intra-thoracic administration of viruses to neonatal and adult mice was performed as previously described^14,23^ using injection of a single bolus (10 ml kg^−1^) via a 30-gauge needle inserted through the diaphragm by a subxiphoid approach into the inferior mediastinum, avoiding the heart and the lung.

### Echocardiographic assessment of mouse heart function and morphology

Serial in vivo echocardiography was performed in lightly anesthetized mice (heart rate ≥500 bpm) with body temperature maintained at 37 °C using Vevo 2100 (FUJIFILM VisualSonics) at 2–4-week intervals as described^12,21^. When anesthesia was discontinued, two-dimensional and M-mode images (parasternal long axis and short axis) of the LV and LA were obtained. Chamber dimensions (LVDd, LVDs, interventricular septal thickness (IVS) and LVPW thickness) were averaged from M-mode tracings from three consecutive heartbeats. Echocardiographic measurements were performed by an experienced observer blinded to mouse genotype.

### Cardiac histology and assessment of fibrosis

Sections (5 μm) of paraffin-embedded hearts were stained with Masson trichrome and photographed on a light microscope (Keyence) as described^12,14,21^. Fibrotic area and collagen fibers (in percent) were assessed using BZ-II Analyzer software (Keyence) from images taken with ×20 objective lens (BZ-9000, Keyence). Paraffin-embedded hearts were sectioned to obtain short-axis, two-chamber views at a minimum of five levels from the apex to the base; each level was ~50 μm apart. Areas containing valve tissue were manually excluded.

### AAV vector production

AAV vectors were produced by the MEEI/SERI Gene Transfer Vector Core (https://www.vdb-lab.org/vector-core/) as previously described^47^. In brief, vector preparations were generated by polyethylenimine (PEI) (Polysciences, 24765-2) triple transfection of pkAAV2/9, pALD-X80 and the plasmids containing the different transgenes in HEK293 cells (Lonza, and not authenticated) seeded into ten-layer HYPERFlasks using a PEIMax/DNA ratio of 1.375:1 (v/w). Three days after transfection, vectors were harvested from the HYPERFlasks using Benzonase (EMD Millipore, 1016970010) to degrade DNA/RNA. Twenty-four hours after harvesting, the vectors were concentrated by tangential flow filtration and purified by iodixanol gradient ultracentrifugation. Vector titers were calculated by digital droplet PCR according to a previously published protocol^48^.

AAV9 contained cloned plasmids, Cas9-nickase and base editors. Plasmid pAAV-Tnnt-eGFP.RBG^14^ plasmid contains an eGFP transgene expressed under the control of a cardiac troponin T promoter (Tnnt) and a rabbit beta globin (RBG) polyadenylation signal. Plasmid pAAV-CMV-mScarlet.WPRE.bGH (https://www.vdb-lab.org/vector-core/) expresses the mScarlet fluorescent protein under the control of the ubiquitous cytomegalovirus (CMV) promoter and contains a woodchuck hepatitis virus post-transcriptional regulatory element (WPRE) and a bovine growth hormone (bGH) polyadenylation signal.

A variant of *S. pyogenes* Cas9-nickase, SpCas9-NG, was used to target the base editor to the R403Q pathogenic variant. This Cas9-nickase was selected because it recognizes a particular PAM NG site (Fig. 1; the non-canonical NGNG sequence) with high efficiency. A PAM NGNG sequence is present in *Myh6* DNA in SvEv and SvEv/S4 mice as well as the homologous site in humans.

The ABE8e-SpCas9-NG base editor (abbreviated in the text as ABE8e) was cloned into established dual-vector split-intein base editor AAV genome plasmids (Addgene plasmids 137176 and 137177) as described^17,49^. However, we replaced the ubiquitously expressing synthetic Cbh promoter with the cardiomyocyte-specific chicken cardiac troponin T promoter (*Tnnt2*)^14^, encoded on a ~800-bp oligonucleotide (gBlock, Integrated DNA Technologies) and XbaI/AgeI-digested AAV9 plasmids. The two DNA molecules were combined using isothermal assembly in NEBuilder HiFi DNA Assembly Master Mix (New England Biolabs (NEB), E2621S).

The selected protospacer (Fig. 1a) places the target R403Q mutant nucleotide at position 5, which is optimal for adenine base editing^18,19^. This protospacer sequence targeting the pathogenic variant R403Q was cloned into the sgRNA cassette in AAV genome vectors as previously described^17^. AAV genome vectors were digested with BsmBI and ligated with pre-annealed oligos encoding the spacer sequence.

Forward oligo: **CACC** GCCTCAGGTGAAGGTGGGGAA

Reverse oligo: **AAAC** TTCCCCACCTTCACCTGAGG

Overhang nucleotides that mediate proper annealing into the sgRNA expression cassette are bolded. Plasmids were purified in high yield and purity by Aldevron for AAV generation.

### High-throughput sequencing of target genomic loci

High-throughput sequencing of gDNA was performed as previously described^49^. Primers for amplification of the Myh6 R403 locus from mouse genomic DNA were:

mMYH6F: 5′-ACACTCTTTCCCTACACGACGCTCTTCCGATCTNNNNCGTGTCACCATCCAGTTGAAC -3′

mMYH6R: 5′-TGGAGTTCAGACGTGTGCTCTTCCGATCTGGATGGGTGGATCAAGGACAT-3′

Underlined sequences represent adapters for Illumina sequencing. Primers used to amplify off-target loci are provided in Supplementary Table 4. After Illumina barcoding, PCR products were pooled and purified by electrophoresis with a 1% agarose gel using a Monarch DNA Gel Extraction Kit (NEB), eluting with 30 μl of water. DNA concentration was quantified with Qubit dsDNA High Sensitivity Assay Kit (Thermo Fisher Scientific) and sequenced on an Illumina MiSeq instrument (single-end read, 250–300 cycles) according to the manufacturer’s protocols. Alignment of FASTQ files and quantification of editing frequency were performed using CRISPResso2 (ref. ^50^) in batch mode with a window size of 20 nucleotides (nt).

### SpCas9 nuclease purification for experimental off-target identification

SpCas9-NG nuclease was cloned into a pET42b plasmid for bacterial expression using a 6xHis purification tag (Addgene, 194705). BL21 Star DE3 chemically competent cells (Invitrogen, C601003) were transformed with the plasmid and picked into 2×YT media supplemented with 25 µg ml^−1^ kanamycin for overnight growth at 37 °C. The next day, 1 L of pre-warmed 2×YT + 25 µg ml^−1^ kanamycin was inoculated at optical density (OD) 0.03 and shaken at 37 °C for about 3 hours until OD reached 0.8. Culture was cold-shocked in an ice water slurry for 1 hour, after which protein expression was induced by the addition of 1 mM IPTG. Culture was shaken at 16 °C for 16 hours to express protein. Cells were pelleted at 6,000*g* for 20 minutes and stored at −80 °C. The next day, cells were resuspended in 30 ml of cold lysis buffer (1 M NaCl, 100 mM Tris-HCl pH 7.0, 5 mM TCEP and 20% glycerol, with three tablets of complete, EDTA-free protease inhibitor cocktail) (MilliporeSigma, 4693132001). Cells were lysed by sonification at 4 °C for a total treatment of 7.5 minutes, providing time to cool after every 3 seconds of treatment. Cell lysate was clarified for 20 minutes using 20,000*g* centrifugation at 4 °C. Supernatant was collected and added to 1.5 ml of Ni-NTA resin slurry (G-Biosciences, 786-940, pre-washed once with lysis buffer). Protein-bound resin was washed twice with 12 ml of lysis buffer in a gravity column. Protein was eluted in 3 ml of elution buffer (200 mM imidazole, 500 mM NaCl, 100 mM Tris-HCl pH 7.0, 5 mM TCEP and 20% glycerol). Eluted protein was diluted in 40 mL of low salt buffer (100 mM Tris-HCl, pH 7.0, 5 mM TCEP and 20% glycerol) just before loading into a 50-ml ÄKTA Superloop for ion exchange purification on the ÄKTA pure 25 FPLC. Ion exchange chromatography was conducted on a 5-ml GE Healthcare HiTrap SP HP pre-packed column. After washing the column with 15 ml of low salt buffer, the diluted protein was flowed through the column to bind. The column was washed in 15 ml of low salt buffer before being subjected to an increasing gradient to a maximum of 80% high salt buffer (1 M NaCl, 100 mM Tris-HCl pH 7.0, 5 mM TCEP and 20% glycerol) over the course of 50 ml, at a flow rate of 5 ml min^−1^. Then, 1-ml fractions were collected during this ramp to high salt buffer. Peaks were assessed by SDS-PAGE to identify fractions containing the desired protein, which were pooled and concentrated using an Amicon Ultra 15-ml centrifugal filter (100-kDa cutoff). SDS-PAGE stained with InstantBlue (Expedeon, SKU ISB1L) was used to visualize the purity after each step. Concentrated protein was quantified using a BCA assay (Thermo Fisher Scientific, 23227); the final concentration was 86.4 µM.

### Off-target identification by CIRCLE-seq

gDNA from HEK293T cells was isolated using Gentra Puregene Kit (Qiagen) according to the manufacturer’s instructions. CIRCLE-seq was performed as previously described^32,51^. Purified gDNA was sheared with a Covaris S2 instrument to an average length of 300 bp. The fragmented DNA was end-repaired, A-tailed and ligated to a uracil-containing stem-loop adaptor, using KAPA HTP Library Preparation Kit, PCR Free (KAPA Biosystems). Adaptor-ligated DNA was treated with Lambda Exonuclease (NEB) and *Escherichia coli* Exonuclease I (NEB) and then with USER enzyme (NEB) and T4 polynucleotide kinase (NEB). Intramolecular circularization of the DNA was performed with T4 DNA ligase (NEB), and residual linear DNA was degraded by Plasmid-Safe ATP-dependent DNase (Lucigen). In vitro cleavage reactions were performed with 250 ng of Plasmid-Safe-treated circularized DNA, 90 nM of SpCas9-NG protein, Cas9 nuclease buffer (NEB) and 90 nM of synthetic chemically modified, slow-annealed sgRNA (Synthego), in a 100-µl volume. The cleavage reaction was stopped by the addition of 10 µl of Proteinase K (NEB, diluted in water to 200 U ml^−1^). Cleaved products were A-tailed, ligated with a hairpin adaptor (NEB), treated with USER enzyme (NEB) and amplified by PCR with barcoded universal primers NEBNext Multiplex Oligos for Illumina (NEB), using Kapa HiFi Polymerase (KAPA Biosystems). Libraries were sequenced with 150-nt paired-end reads on an Illumina MiSeq instrument. CIRCLE-seq data analyses were performed using open-source CIRCLE-seq analysis software and default recommended parameters (https://github.com/tsailabSJ/circleseq). We selected the top 16 off-target sites at which the greatest number of read counts mapped to analyze, but most of these were in repetitive elements and could not be specifically PCR amplified. We then sequenced the top 16 sites that did not share the sequence signature of the repetitive element and successfully amplified and measured editing at these sites.

### Analyses of total RNA by RNA-seq and Myh6 RNA by RT–PCR

Total RNA for RNA-seq analyses and for analysis of Myh6 RNA and snRNA-seq was prepared as described previously^14,31^. Mouse hearts were washed in ice-cold PBS to remove contaminating blood. Left ventricular tissue from mice (ages 42–56 weeks) was collected for RNA extraction. Tissue was homogenized in TRIzol Reagent (Life Technologies) with TissueLyzer (Qiagen), and RNA was extracted by conventional methods. Preparation of libraries and sequencing was described in detail previously^14,21,31^. In brief, two rounds of mRNA purification (polyA selection) were performed on 2 μg of total RNA using Dynabeads mRNA DIRECT Kit (Invitrogen). cDNA was generated using the Superscript III First-Strand Synthesis System (Invitrogen), and the subsequent cDNA libraries were constructed using the Nextera XT DNA Sample Preparation Kit (Illumina). Libraries were sequenced on the Illumina HiSeq 2500. Data were normalized to the total number of reads per kilobase of exon per million (RPKM). Reads were aligned to mouse genome sequence (version mm10 (GRCm38) (GenBank ID 326478)) using Spliced Transcripts Alignment to a Reference (STAR).

The relative numbers of mutant (R403Q) and WT R403 sequence (Fig. 1a) were evaluated using RT–PCR as described previously^14^ (primer positions shown in Extended Data Fig. 2). In brief, ~100 ng of total RNA was reverse transcribed using an Myh6-specific primer (Extended Data Fig. 2): RTprimer_Myh6- GCCTTTGATGTGCTGAGCTT

Twenty percent of the subsequent reverse transcriptase product was PCR amplified by two independent primer pairs:

Primer pair 1

Chr14_Ex13_1_1 (Forward): AGCCTGATGGCACAGAAG Chr14_Ex13_1_2 (Reverse): CAAAGCTGTTGAAATCGAAGA

Primer pair 2

Chr14_Ex13_2_1 (Forward): CCATCATGCACTACGGAAAC Chr14_Ex13_2_2 (Reverse): CTGCAGCTTCTCATTGGTGA

Amplified PCR product was converted into a Nextera sequencing library and sequenced on an Illumina MiSeq. Approximately 100,000 reads were obtained per amplified fragment and aligned to the mouse mm10 genome. The numbers of R403, R403Q (Fig. 1a; position 5 A/G) and bystander (Fig. 1a; A10 and A11) containing reads were counted using a custom R script. The script employed the formula:

Percent of R403Q allele inactivation = (1 – (total number of R403Q reads, divided by total number of WT reads)) multiplied by 100.

### snRNA-seq

snRNA-seq was performed as previously described^52^. In summary, nuclei were extracted from excised and snap-frozen tissues using a TissueLyzer (Qiagen) and homogenization buffer (250 mM sucrose, 25 mM KCl, 5 mM MgCl_2_, 10 mM Tris-HCL, 1 mM dithiothreitol (DTT) and 1× protease inhibitor), The homogenate was filtered through a 40-µm strainer (Corning). After centrifugation (500*g*, 5 minutes, 4 °C), the supernatant was removed, and the pellet was resuspended in storage buffer (1× PBS, 4% BSA, 0.2 U µl^−1^ Protector RNaseIn). Nuclei were stained with NucBlue Live ReadyProbes Reagent (Thermo Fisher Scientific), and Hoechst-positive nuclei were purified by fluorescence-activated cell sorting (FACS) (Aria, BD Biosciences). Intact nuclei were further fractionated on the Chromium Controller (10x Genomics) according to the manufacturer’s protocol with a targeted nuclei recovery of 8,000 per run. 3′ gene expression libraries were prepared according to the manufacturer’s instructions of the version 3.1 Chromium Single Cell Reagent Kits (10x Genomics). Libraries were sequenced on a NovaSeq 6000 (Illumina) with a minimum depth of 20,000–30,000 read pairs per nucleus. Reads were aligned to a modified mm10 mouse genome using Cell Ranger (version 3.0.1) to account for the AAV9 genome plasmid. Harmony was used to reduce the contribution of batch effects^53,54^. Louvain clustering and uniform manifold approximation and projection (UMAP) visualization were performed to identify cell types.

### Analysis of transcriptome-wide off-target RNA base editing

Analysis of transcriptome-wide off-target RNA base editing was performed as previously described^20^. In brief, REDItools version 1.3 (ref. ^55^) was used to quantify the average percentage of A-to-I editing among all sequenced adenosines in each sample. Any adenines with a read depth less than 10 or a read quality score below 30 were removed from the analysis. The transcriptome-wide A-to-I editing frequency was calculated as: (number of reads in which an adenosine was called as a guanosine) divided by (total number of reads covering all analyzed adenosines).

### Reporting Summary

Further information on research design is available in the Nature Portfolio Reporting Summary linked to this article.

## Online content

Any methods, additional references, Nature Portfolio reporting summaries, source data, extended data, supplementary information, acknowledgements, peer review information; details of author contributions and competing interests; and statements of data and code availability are available at 10.1038/s41591-022-02190-7.

## Supplementary information

Supplementary Information Supplementary Information contains sequences for editor open reading frames, *SaCas9* nuclease and sgRNAs; Extended Data figure legends; and Supplementary Table legends, and it lists the Supplementary Tables and Supplementary Data Source Files for figures and Extended Data figures.

Reporting Summary

Supplementary Tables 1–5 (single workbook).

## Data Availability

Fluorescent plasmids are available through the MEEI/SERI Gene Transfer Vector Core (https://www.vdb-lab.org/vector-core/). All others have been deposited on Addgene (https://www.addgene.org/). Mouse genome sequences (version mm10 (GRCm38)) are available at https://www.genome.ucsc.edu. Sequencing read data are available through the National Center for Biotechnology Information Gene Expression Omnibus database (Superseries accession number GSE220851; individual accession numbers GSE220517, GSE220808 and GSE220811). There are no restrictions on the use of these data. Source data are provided with this paper.

## Source data

Source Data Fig. 1 Source Data for Fig. 1
Source Data Fig. 2 Source Data for Fig. 2
Source Data Fig. 3 Source Data for Fig. 3
Source Data Fig. 4 Source Data for Fig. 4
Source Data Fig. 5 Source Data for Fig. 5
Source Data Extended Data Fig. 4 Source Data for Extended Data Fig. 4
Source Data Extended Data Fig.6 Source Data for Extended Data Fig. 6
Source Data Extended Data Fig. 7 Source Data for Extended Data Fig. 7
Source Data Extended Data Fig. 8 Source Data for Extended Data Fig. 8
Source Data Extended Data Fig. 9 Source Data for Extended Data Fig. 9
Source Data Extended Data Fig. 10 Source Data for Extended Data Fig. 10
