# Supplementary material for: Efficient in vivo genome editing prevents hypertrophic cardiomyopathy in mice
Source: Nat Med. 2023 Feb 16;29(2):412–21. doi: 10.1038/s41591-022-02190-7 (PMC9941048; doi:10.1038/s41591-022-02190-7)
Supplement: Supplementary file 1 — Supplementary Information contains sequences for editor open reading frames, SaCas9 nuclease and sgRNAs; Extended Data figure legends; and Supplementary Table legends, and it lists the Supplementary Tables and Supplementary Data Source Files for figures and Extended Data figures. [file 41591_2022_2190_MOESM1_ESM.pdf]

nature medicine

Article

https://doi.org/10.1038/s41591-022-02190-7

# Efficient in vivo genome editing prevents hypertrophic cardiomyopathy in mice

In the format provided by the authors and unedited

**Title:** ***Efficient in vivo Genome Editing Prevents Hypertrophic Cardiomyopathy in Mice***

**Authors:** Daniel Reichart[1,2^], Gregory A. Newby[3,4,5^], Hiroko Wakimoto[1^], Mingyue Lun[1], Joshua M. Gorham[1], Justin J. Curran[1], Aditya Raguram[3,4,5], Daniel M. DeLaughter[1,5], David A. Conner[1], Júlia D. C. Marsiglia[1], Sajeev Kohli[3,4,5], Lukas Chmatal[6], David C. Page[5,6,7], Nerea Zabaleta[8,9,10], Luk Vandenberghe[8,9,10], David R. Liu[3,4,5*], Jonathan G. Seidman[1*], and Christine Seidman[1,5,11*]

**Affiliations:**

1. Genetics Department, Harvard Medical School, Boston, MA
2. Department of Medicine I, University Hospital, LMU Munich, Munich, Germany
3. Merkin Institute of Transformative Technologies in Healthcare, Broad Institute of Harvard and MIT, Cambridge, MA
4. Department of Chemistry and Chemical Biology, Harvard University, Cambridge, MA
5. Howard Hughes Medical Institute; Chevy Chase, MD
6. Whitehead Institute, Cambridge, MA
7. Department of Biology, Massachusetts Institute of Technology, Cambridge, MA
8. Grousbeck Gene Therapy Center, Schepens Eye Research Institute, Mass Eye and Ear, Boston, MA
9. Ocular Genomics Institute, Department of Ophthalmology, Harvard Medical School, Boston, MA
10. Harvard Stem Cell Institute, Harvard University, Cambridge, MA
11. Cardiovascular Division, Brigham and Women's Hospital; Boston, MA

## Inventory of Supporting Information

Sequences for editor ORFs, *SaCas9* nuclease, and sgRNAs Page 2

Supplementary Table Legends Page 10

Supplementary Tables 1-5 (.xls)

Supplementary Data Source Files for Figs. 1-5 and Extended Data Figs. 4, 6-10 (.xls)

## Supplementary Information

*ABE8e-NG N-terminal vector ORF sequence (NpuN intein fusion at C-terminus):*

ATGAAACGGACAGCCGACGGAAGCGAGTTCGAGTCACCAAAGAAGAAGCGGAAAGTCTCT
GAGGTGGAGTTTTCCCACGAGTACTGGATGAGACATGCCCTGACCCTGGCCAAGAGGGCA
CGGGATGAGAGGGAGGTGCCTGTGGGAGCCGTGCTGGTGCTGAACAATAGAGTGATCGG
CGAGGGCTGGAACAGAGCCATCGGCCTGCACGACCCAACAGCCCATGCCGAAATTATGG
CCCTGAGACAGGGCGGCCTGGTCATGCAGAACTACAGACTGATTGACGCCACCCTGTACG
TGACATTCGAGCCTTGCGTGATGTGCGCCGGCGCCATGATCCACTCTAGGATCGGCCGCG
TGGTGTTTGGCGTGAGGAACTCAAAAAGAGGCGCCGCAGGCTCCCTGATGAACGTGCTGA
ACTACCCCGGCATGAATCACCGCGTCGAAATTACCGAGGGAATCCTGGCAGATGAATGTG
CCGCCCTGCTGTGCGATTTCTATCGGATGCCTAGACAGGTGTTCAATGCTCAGAAGAAGG
CCCAGAGCTCCATCAACTCCGGAGGATCTAGCGGAGGCTCCTCTGGCTCTGAGACACCTG
GCACAAGCGAGAGCGCAACACCTGAAAGCAGCGGGGGCAGCAGCGGGGGGTCAGACAA
GAAGTACAGCATCGGCCTGGCCATCGGCACCAACTCTGTGGGCTGGGCCGTGATCACCG
ACGAGTACAAGGTGCCCAGCAAGAAATTCAAGGTGCTGGGCAACACCGACCGGCACAGCA
TCAAGAAGAACCTGATCGGAGCCCTGCTGTTCGACAGCGGCGAAACAGCCGAGGCCACC
CGGCTGAAGAGAACCGCCAGAAGAAGATACACCAGACGGAAGAACCGGATCTGCTATCTG
CAAGAGATCTTCAGCAACGAGATGGCCAAGGTGGACGACAGCTTCTTCCACAGACTGGAA
GAGTCCTTCCTGGTGGAAGAGGATAAGAAGCACGAGCGGCACCCCATCTTCGGCAACATC
GTGGACGAGGTGGCCTACCACGAGAAGTACCCCACCATCTACCACCTGAGAAAGAAACTG
GTGGACAGCACCGACAAGGCCGACCTGCGGCTGATCTATCTGGCCCTGGCCCACATGATC
AAGTTCCGGGGCCACTTCCTGATCGAGGGCGACCTGAACCCCGACAACAGCGACGTGGA
CAAGCTGTTCATCCAGCTGGTGCAGACCTACAACCAGCTGTTCGAGGAAAACCCCATCAAC
GCCAGCGGCGTGGACGCCAAGGCCATCCTGTCTGCCAGACTGAGCAAGAGCAGACGGCT
GGAAAATCTGATCGCCCAGCTGCCCGGCGAGAAGAAGAATGGCCTGTTCGGAAACCTGAT
TGCCCTGAGCCTGGGCCTGACCCCCAACTTCAAGAGCAACTTCGACCTGGCCGAGGATGC
CAAACTGCAGCTGAGCAAGGACACCTACGACGACGACCTGGACAACCTGCTGGCCCAGAT
CGGCGACCAGTACGCCGACCTGTTTCTGGCCGCCAAGAACCTGTCCGACGCCATCCTGCT
GAGCGACATCCTGAGAGTGAACACCGAGATCACCAAGGCCCCCCTGAGCGCCTCTATGAT
CAAGAGATACGACGAGCACCACCAGGACCTGACCCTGCTGAAAGCTCTCGTGCGGCAGCA
GCTGCCTGAGAAGTACAAAGAGATTTTCTTCGACCAGAGCAAGAACGGCTACGCCGGCTA
CATTGACGGCGGAGCCAGCCAGGAAGAGTTCTACAAGTTCATCAAGCCCATCCTGGAAAA
GATGGACGGCACCGAGGAACTGCTCGTGAAGCTGAACAGAGAGGACCTGCTGCGGAAGC

AGCGGACCTTCGACAACGGCAGCATCCCCCACCAGATCCACCTGGGAGAGCTGCACGCC
ATTCTGCGGCGGCAGGAAGATTTTTACCCATTCCTGAAGGACAACCGGGAAAAGATCGAG
AAGATCCTGACCTTCCGCATCCCCTACTACGTGGGCCCTCTGGCCAGGGGAAACAGCAGA
TTCGCCTGGATGACCAGAAAGAGCGAGGAAACCATCACCCCCTGGAACTTCGAGGAAGTG
GTGGACAAGGGCGCTTCCGCCCAGAGCTTCATCGAGCGGATGACCAACTTCGATAAGAAC
CTGCCCAACGAGAAGGTGCTGCCCAAGCACAGCCTGCTGTACGAGTACTTCACCGTGTAT
AACGAGCTGACCAAAGTGAAATACGTGACCGAGGGAATGAGAAAGCCCGCCTTCCTGAGC
GGCGAGCAGAAAAAGGCCATCGTGGACCTGCTGTTCAAGACCAACCGGAAAGTGACCGTG
AAGCAGCTGAAAGAGGACTACTTCAAGAAAATCGAGTGCCTGTCCTACGAGACAGAGATC
CTGACAGTGGAGTATGGCCTGCTGCCAATCGGCAAGATCGTGGAGAAGAGGATCGAGTGT
ACCGTGTACTCTGTGGATAACAATGGCAACATCTATACACAGCCCGTGGCACAGTGGCAC
GATAGGGGAGAGCAGGAGGTGTTCGAGTATTGCCTGGAGGACGGCAGCCTGATCAGGGC
AACCAAGGACCACAAGTTCATGACAGTGGATGGCCAGATGCTGCCCATCGACGAGATTTT
CGAGCGGGAGCTGGACCTGATGAGAGTGGATAACCTGCCTAATAG

*ABE sgRNA sequence* (Note: the nucleotide that displaces the disease-causing nucleotide we target for editing is underlined and the scaffold is bolded):

GCCUCAGGUGAAGGUGGGAA**GUUUUAGAGCUAGAAAUAGCAAGUUAAAAUAAGGCU
AGUCCGUUAUCAACUUGAAAAAGUGGCACCGAGUCGGUGC**

*ABE8e-NG C-terminal vector ORF sequence (NpuC intein fusion at N-terminus):*

ATGAAACGGACAGCCGACGGAAGCGAGTTCGAGTCACCAAAGAAGAAGCGGAAAGTCATC
AAGATTGCTACACGGAAATACCTGGGAAAGCAGAACGTGTACGACATCGGCGTGGAGCGG
GATCACAACTTCGCCCTGAAGAATGGCTTTATCGCCAGCAATTGCTTCGACTCCGTGGAAA
TCTCCGGCGTGGAAGATCGGTTCAACGCCTCCCTGGGCACATACCACGATCTGCTGAAAA
TTATCAAGGACAAGGACTTCCTGGACAATGAGGAAAACGAGGACATTCTGGAAGATATCGT
GCTGACCCTGACACTGTTTGAGGACAGAGAGATGATCGAGGAACGGCTGAAAACCTATGC
CCACCTGTTCGACGACAAAGTGATGAAGCAGCTGAAGCGGCGGAGATACACCGGCTGGG
GCAGGCTGAGCCGGAAGCTGATCAACGGCATCCGGGACAAGCAGTCCGGCAAGACAATC
CTGGATTTCCTGAAGTCCGACGGCTTCGCCAACAGAAACTTCATGCAGCTGATCCACGAC
GACAGCCTGACCTTTAAAGAGGACATCCAGAAAGCCCAGGTGTCCGGCCAGGGCGATAGC
CTGCACGAGCACATTGCCAATCTGGCCGGCAGCCCCGCCATTAAGAAGGGCATCCTGCAG
ACAGTGAAGGTGGTGGACGAGCTCGTGAAAGTGATGGGCCGGCACAAGCCCGAGAACAT
CGTGATCGAAATGGCCAGAGAGAACCAGACCACCCAGAAGGGACAGAAGAACAGCCGCG

AGAGAATGAAGCGGATCGAAGAGGGCATCAAAGAGCTGGGCAGCCAGATCCTGAAAGAAC
ACCCCGTGGAAAACACCCAGCTGCAGAACGAGAAGCTGTACCTGTACTACCTGCAGAATG
GGCGGGATATGTACGTGGACCAGGAACTGGACATCAACCGGCTGTCCGACTACGATGTGG
ACCATATCGTGCCTCAGAGCTTTCTGAAGGACGACTCCATCGACAACAAGGTGCTGACCA
GAAGCGACAAGAACCGGGGCAAGAGCGACAACGTGCCCTCCGAAGAGGTCGTGAAGAAG
ATGAAGAACTACTGGCGGCAGCTGCTGAACGCCAAGCTGATTACCCAGAGAAAGTTCGAC
AATCTGACCAAGGCCGAGAGAGGCGGCCTGAGCGAACTGGATAAGGCCGGCTTCATTAAG
AGACAGCTGGTGGAAACCCGGCAGATCACAAAGCACGTGGCACAGATCCTGGACTCCCG
GATGAACACTAAGTACGACGAGAATGACAAGCTGATCCGGGAAGTGAAAGTGATCACCCT
GAAGTCCAAGCTGGTGTCCGATTTCCGGAAGGATTTCCAGTTTTACAAAGTGCGCGAGATC
AACAACTACCACCACGCCCACGACGCCTACCTGAACGCCGTCGTGGGAACCGCCCTGATC
AAAAAGTACCCTAAGCTGGAAAGCGAGTTCGTGTACGGCGACTACAAGGTGTACGACGTG
CGGAAGATGATCGCCAAGAGCGAGCAGGAAATCGGCAAGGCTACCGCCAAGTACTTCTTC
TACAGCAACATCATGAACTTTTTCAAGACCGAGATTACCCTGGCCAACGGCGAGATCCGGA
AGCGGCCTCTGATCGAGACAAACGGCGAAACCGGGGAGATCGTGTGGGATAAGGGCCGG
GATTTTGCCACCGTGCGGAAAGTGCTGAGCATGCCCCAAGTGAATATCGTGAAAAAGACC
GAGGTGCAGACAGGCGGCTTCAGCAAAGAGTCTATCCGGCCCAAGAGGAACAGCGATAA
GCTGATCGCCAGAAAGAAGGACTGGGACCCTAAGAAGTACGGCGGCTTCGTGAGCCCCA
CCGTGGCCTATTCTGTGCTGGTGGTGGCCAAAGTGGAAAAGGGCAAGTCCAAGAAACTGA
AGAGTGTGAAAGAGCTGCTGGGGATCACCATCATGGAAAGAAGCAGCTTCGAGAAGAATC
CCATCGACTTTCTGGAAGCCAAGGGCTACAAAGAAGTGAAAAAGGACCTGATCATCAAGCT
GCCTAAGTACTCCCTGTTCGAGCTGGAAAACGGCCGGAAGAGAATGCTGGCCTCTGCCCG
GTTCCTGCAGAAGGGAAACGAACTGGCCCTGCCCTCCAAATATGTGAACTTCCTGTACCTG
GCCAGCCACTATGAGAAGCTGAAGGGCTCCCCCGAGGATAATGAGCAGAAACAGCTGTTT
GTGGAACAGCACAAGCACTACCTGGACGAGATCATCGAGCAGATCAGCGAGTTCTCCAAG
AGAGTGATCCTGGCCGACGCTAATCTGGACAAAGTGCTGTCCGCCTACAACAAGCACCGG
GATAAGCCCATCAGAGAGCAGGCCGAGAATATCATCCACCTGTTTACCCTGACCAATCTGG
GAGCCCCTCGGGCCTTCAAGTACTTTGACACCACCATCGACCGGAAGGTGTACCGGAGCA
CCAAAGAGGTGCTGGACGCCACCCTGATCCACCAGAGCATCACCGGCCTGTACGAGACAC
GGATCGACCTGTCTCAGCTGGGAGGTGACTCTGGCGGCTCAAAAAGAACCGCCGACGGC
AGCGAATTCGAGCCCAAGAAGAAGAGGAAAGTCTAA

*SaCas9 nuclease sgRNA sequence* (Note: the allele-specific nucleotide is underlined and the scaffold is bolded):

GUACUCAUUGCCCACUUUCACCU**GUUUUAGUACUCUGUAAUGAAAAUUACAGAAUCUA**
**CUAAAACAAGGCAAAAUGCCGUGUUUAUCUCGUCAACUUGUUGGCGAGAUUU**

*SaCas9 nuclease ORF sequence:*

ATGAAACGGACAGCCGACGGAAGCGAGTTCGAGTCACCAAAGAAGAAGCGGAAAGTCGG
GAAGCGAAATTACATTCTGGGGCTGGATATTGGCATTACATCAGTGGGCTATGGCATCATT
GACTACGAGACAAGGGACGTGATCGACGCCGGCGTGAGACTGTTCAAGGAGGCCAACGT
GGAGAACAATGAGGGCCGGAGATCCAAGAGGGGAGCAAGGCGCCTGAAGCGGAGAAGG
CGCCACAGAATCCAGAGAGTGAAGAAGCTGCTGTTCGATTACAACCTGCTGACCGACCAC
TCCGAGCTGTCTGGCATCAATCCTTATGAGGCCAGAGTGAAGGGCCTGTCCCAGAAGCTG
TCTGAGGAGGAGTTTAGCGCCGCCCTGCTGCACCTGGCAAAGAGGAGAGGCGTGCACAA
CGTGAATGAGGTGGAGGAGGACACCGGCAACGAGCTGTCCACAAAGGAGCAGATCAGCC
GCAATTCCAAGGCCCTGGAGGAGAAGTATGTGGCCGAGCTGCAGCTGGAGCGGCTGAAG
AAGGATGGCGAGGTGAGGGGCTCCATCAATCGCTTCAAGACCTCTGACTACGTGAAGGAG
GCCAAGCAGCTGCTGAAGGTGCAGAAGGCCTACCACCAGCTGGATCAGTCCTTTATCGAT
ACATATATCGACCTGCTGGAGACAAGGCGCACATACTATGAGGGACCAGGAGAGGGCTCT
CCCTTCGGCTGGAAGGACATCAAGGAGTGGTACGAGATGCTGATGGGCCACTGCACCTAT
TTTCCAGAGGAGCTGAGAAGCGTGAAGTACGCCTATAACGCCGATCTGTACAACGCCCTG
AATGACCTGAACAACCTGGTCATCACCAGGGATGAGAACGAGAAGCTGGAGTACTATGAG
AAGTTCCAGATCATCGAGAACGTGTTCAAGCAGAAGAAGAAGCCTACACTGAAGCAGATCG
CCAAGGAGATCCTGGTGAACGAGGAGGACATCAAGGGCTACCGCGTGACCTCCACAGGC
AAGCCAGAGTTCACCAATCTGAAGGTGTATCACGATATCAAGGACATCACAGCCCGGAAG
GAGATCATCGAGAACGCCGAGCTGCTGGATCAGATCGCCAAGATCCTGACCATCTATCAG
AGCTCCGAGGACATCCAGGAGGAGCTGACCAACCTGAATAGCGAGCTGACACAGGAGGA
GATCGAGCAGATCAGCAATCTGAAGGGCTACACCGGCACACACAACCTGAGCCTGAAGGC
CATCAATCTGATCCTGGATGAGCTGTGGCACACAAACGACAATCAGATCGCCATCTTTAAC
CGGCTGAAGCTGGTGCCAAAGAAGGTGGACCTGTCCCAGCAGAAGGAGATCCCAACCAC
ACTGGTGGACGATTTCATCCTGTCTCCCGTGGTGAAGCGGAGCTTCATCCAGAGCATCAAA
GTGATCAACGCCATCATCAAGAAGTACGGCCTGCCCAATGATATCATCATCGAGCTGGCCA
GGGAGAAGAACTCCAAGGACGCCCAGAAGATGATCAATGAGATGCAGAAGAGGAACCGC
CAGACCAATGAGCGGATCGAGGAGATCATCAGAACCACAGGCAAGGAGAACGCCAAGTAC
CTGATCGAGAAGATCAAGCTGCACGATATGCAGGAGGGCAAGTGTCTGTATTCTCTGGAG
GCCATCCCTCTGGAGGACCTGCTGAACAATCCATTCAACTACGAGGTGGATCACATCATCC
CCCGGAGCGTGAGCTTCGACAATTCTTTTAACAATAAGGTGCTGGTGAAGCAGGAGGAGA

ACAGCAAGAAGGGCAATAGGACCCCTTTCCAGTACCTGTCTAGCTCCGATTCTAAGATCAG
CTACGAGACATTCAAGAAGCACATCCTGAATCTGGCCAAGGGCAAGGGCCGCATCAGCAA
GACCAAGAAGGAGTACCTGCTGGAGGAGCGGGACATCAACAGATTCTCCGTGCAGAAGGA
CTTCATCAACCGGAATCTGGTGGACACCAGATACGCCACACGCGGCCTGATGAATCTGCT
GCGGTCTTATTTCAGAGTGAACAATCTGGATGTGAAGGTGAAGAGCATCAACGGCGGCTT
CACCTCCTTTCTGCGGAGAAAGTGGAAGTTTAAGAAGGAGCGCAACAAGGGCTATAAGCA
CCACGCCGAGGATGCCCTGATCATCGCCAATGCCGACTTCATCTTTAAGGAGTGGAAGAA
GCTGGACAAGGCCAAGAAAGTGATGGAGAACCAGATGTTCGAGGAGAAGCAGGCCGAGA
GCATGCCCGAGATCGAGACAGAGCAGGAGTACAAGGAGATTTTCATCACACCTCACCAGA
TCAAGCACATCAAGGACTTCAAGGACTACAAGTATTCTCACAGGGTGGATAAGAAGCCCAA
CCGCGAGCTGATCAATGACACCCTGTATAGCACACGGAAGGACGATAAGGGCAATACCCT
GATCGTGAACAATCTGAACGGCCTGTACGACAAGGATAATGACAAGCTGAAGAAGCTGATC
AACAAGTCTCCCGAGAAGCTGCTGATGTACCACCACGATCCTCAGACATATCAGAAGCTGA
AGCTGATCATGGAGCAGTACGGCGACGAGAAGAACCCACTGTATAAGTACTATGAGGAGA
CAGGCAACTACCTGACAAAGTATAGCAAGAAGGATAATGGCCCCGTGATCAAGAAGATCAA
GTACTATGGCAACAAGCTGAATGCCCACCTGGACATCACCGACGATTACCCTAACTCTCGC
AATAAGGTGGTGAAGCTGAGCCTGAAGCCATACCGGTTCGACGTGTACCTGGACAACGGC
GTGTATAAGTTTGTGACAGTGAAGAATCTGGATGTGATCAAGAAGGAGAACTACTATGAGG
TGAACAGCAAGTGCTACGAGGAGGCCAAGAAGCTGAAGAAGATCAGCAACCAGGCCGAGT
TCATCGCCTCTTTTTACAACAATGACCTGATCAAGATCAATGGCGAGCTGTATAGAGTGATC
GGCGTGAACAATGATCTGCTGAACAGAATCGAAGTGAATATGATCGACATCACCTACAGGG
AGTATCTGGAGAACATGAATGATAAGAGGCCCCCTCGCATCATCAAGACCATCGCCTCTAA
GACACAGAGCATCAAGAAGTACAGCACAGACATCCTGGGGAACCTGTATGAAGTCAAGAG
CAAGAAACATCCTCAGATTATCAAGAAAGGCTCTGGCGGCTCAAAAAGAACCGCCGACGG
CAGCGAATTCGAGCCCAAGAAGAAGAGGAAAGTCTAAA

*SpCas9-NG nuclease ORF Sequence:*

ATGGGCAGCAGTCATCATCATCACCATCACGACAAGAAGTACAGCATCGGCCTGG
ACATCGGCACCAACTCTGTGGGCTGGGCCGTGATCACCGACGAGTACAAGGTGC
CCAGCAAGAAATTCAAGGTGCTGGGCAACACCGACCGGCACAGCATCAAGAAGAA
CCTGATCGGAGCCCTGCTGTTCGACAGCGGCGAAACAGCCGAGGCCACCCGGCT
GAAGAGAACCGCCAGAAGAAGATACACCAGACGGAAGAACCGGATCTGCTATCTG
CAAGAGATCTTCAGCAACGAGATGGCCAAGGTGGACGACAGCTTCTTCCACAGAC
TGGAAGAGTCCTTCCTGGTGGAAGAGGATAAGAAGCACGAGCGGCACCCCATCTT

CGGCAACATCGTGGACGAGGTGGCCTACCACGAGAAGTACCCCACCATCTACCAC
CTGAGAAAGAAACTGGTGGACAGCACCGACAAGGCCGACCTGCGGCTGATCTATC
TGGCCCTGGCCCACATGATCAAGTTCCGGGGCCACTTCCTGATCGAGGGCGACCT
GAACCCCGACAACAGCGACGTGGACAAGCTGTTCATCCAGCTGGTGCAGACCTAC
AACCAGCTGTTCGAGGAAAACCCCATCAACGCCAGCGGCGTGGACGCCAAGGCC
ATCCTGTCTGCCAGACTGAGCAAGAGCAGACGGCTGGAAAATCTGATCGCCCAGC
TGCCCGGCGAGAAGAAGAATGGCCTGTTCGGAAACCTGATTGCCCTGAGCCTGG
GCCTGACCCCCAACTTCAAGAGCAACTTCGACCTGGCCGAGGATGCCAAACTGCA
GCTGAGCAAGGACACCTACGACGACGACCTGGACAACCTGCTGGCCCAGATCGG
CGACCAGTACGCCGACCTGTTTCTGGCCGCCAAGAACCTGTCCGACGCCATCCTG
CTGAGCGACATCCTGAGAGTGAACACCGAGATCACCAAGGCCCCCCTGAGCGCCT
CTATGATCAAGAGATACGACGAGCACCACCAGGACCTGACCCTGCTGAAAGCTCT
CGTGCGGCAGCAGCTGCCTGAGAAGTACAAAGAGATTTTCTTCGACCAGAGCAAG
AACGGCTACGCCGGCTACATTGACGGCGGAGCCAGCCAGGAAGAGTTCTACAAGT
TCATCAAGCCCATCCTGGAAAAGATGGACGGCACCGAGGAACTGCTCGTGAAGCT
GAACAGAGAGGACCTGCTGCGGAAGCAGCGGACCTTCGACAACGGCAGCATCCC
CCACCAGATCCACCTGGGAGAGCTGCACGCCATTCTGCGGCGGCAGGAAGATTTT
TACCCATTCCTGAAGGACAACCGGGAAAAGATCGAGAAGATCCTGACCTTCCGCA
TCCCCTACTACGTGGGCCCTCTGGCCAGGGGAAACAGCAGATTCGCCTGGATGAC
CAGAAAGAGCGAGGAAACCATCACCCCCTGGAACTTCGAGGAAGTGGTGGACAAG
GGCGCTTCCGCCCAGAGCTTCATCGAGCGGATGACCAACTTCGATAAGAACCTGC
CCAACGAGAAGGTGCTGCCCAAGCACAGCCTGCTGTACGAGTACTTCACCGTGTA
TAACGAGCTGACCAAAGTGAAATACGTGACCGAGGGAATGAGAAAGCCCGCCTTC
CTGAGCGGCGAGCAGAAAAAGGCCATCGTGGACCTGCTGTTCAAGACCAACCGG
AAAGTGACCGTGAAGCAGCTGAAAGAGGACTACTTCAAGAAAATCGAGTGCTTCG
ACTCCGTGGAAATCTCCGGCGTGGAAGATCGGTTCAACGCCTCCCTGGGCACATA
CCACGATCTGCTGAAAATTATCAAGGACAAGGACTTCCTGGACAATGAGGAAAACG
AGGACATTCTGGAAGATATCGTGCTGACCCTGACACTGTTTGAGGACAGAGAGAT
GATCGAGGAACGGCTGAAAACCTATGCCCACCTGTTCGACGACAAAGTGATGAAG
CAGCTGAAGCGGCGGAGATACACCGGCTGGGGCAGGCTGAGCCGGAAGCTGATC
AACGGCATCCGGGACAAGCAGTCCGGCAAGACAATCCTGGATTTCCTGAAGTCCG

ACGGCTTCGCCAACAGAAACTTCATGCAGCTGATCCACGACGACAGCCTGACCTT
TAAAGAGGACATCCAGAAAGCCCAGGTGTCCGGCCAGGGCGATAGCCTGCACGA
GCACATTGCCAATCTGGCCGGCAGCCCCGCCATTAAGAAGGGCATCCTGCAGACA
GTGAAGGTGGTGGACGAGCTCGTGAAAGTGATGGGCCGGCACAAGCCCGAGAAC
ATCGTGATCGAAATGGCCAGAGAGAACCAGACCACCCAGAAGGGACAGAAGAACA
GCCGCGAGAGAATGAAGCGGATCGAAGAGGGCATCAAAGAGCTGGGCAGCCAGA
TCCTGAAAGAACACCCCGTGGAAAACACCCAGCTGCAGAACGAGAAGCTGTACCT
GTACTACCTGCAGAATGGGCGGGATATGTACGTGGACCAGGAACTGGACATCAAC
CGGCTGTCCGACTACGATGTGGACCATATCGTGCCTCAGAGCTTTCTGAAGGACG
ACTCCATCGACAACAAGGTGCTGACCAGAAGCGACAAGAACCGGGGCAAGAGCG
ACAACGTGCCCTCCGAAGAGGTCGTGAAGAAGATGAAGAACTACTGGCGGCAGCT
GCTGAACGCCAAGCTGATTACCCAGAGAAAGTTCGACAATCTGACCAAGGCCGAG
AGAGGCGGCCTGAGCGAACTGGATAAGGCCGGCTTCATCAAGAGACAGCTGGTG
GAAACCCGGCAGATCACAAAGCACGTGGCACAGATCCTGGACTCCCGGATGAACA
CTAAGTACGACGAGAATGACAAGCTGATCCGGGAAGTGAAAGTGATCACCCTGAA
GTCCAAGCTGGTGTCCGATTTCCGGAAGGATTTCCAGTTTTACAAAGTGCGCGAGA
TCAACAACTACCACCACGCCCACGACGCCTACCTGAACGCCGTCGTGGGAACCGC
CCTGATCAAAAAGTACCCTAAGCTGGAAAGCGAGTTCGTGTACGGCGACTACAAG
GTGTACGACGTGCGGAAGATGATCGCCAAGAGCGAGCAGGAAATCGGCAAGGCT
ACCGCCAAGTACTTCTTCTACAGCAACATCATGAACTTTTTCAAGACCGAGATTACC
CTGGCCAACGGCGAGATCCGGAAGCGGCCTCTGATCGAGACAAACGGCGAAACC
GGGGAGATCGTGTGGGATAAGGGCCGGGATTTTGCCACCGTGCGGAAAGTGCTG
AGCATGCCCCAAGTGAATATCGTGAAAAAGACCGAGGTGCAGACAGGCGGCTTCA
GCAAAGAGTCTATCCGGCCCAAGAGGAACAGCGATAAGCTGATCGCCAGAAAGAA
GGACTGGGACCCTAAGAAGTACGGCGGCTTCGTGAGCCCCACCGTGGCCTATTCT
GTGCTGGTGGTGGCCAAAGTGGAAAAGGGCAAGTCCAAGAAACTGAAGAGTGTGA
AAGAGCTGCTGGGGATCACCATCATGGAAAGAAGCAGCTTCGAGAAGAATCCCAT
CGACTTTCTGGAAGCCAAGGGCTACAAAGAAGTGAAAAAGGACCTGATCATCAAG
CTGCCTAAGTACTCCCTGTTCGAGCTGGAAAACGGCCGGAAGAGAATGCTGGCCT
CTGCCCGGTTCCTGCAGAAGGGAAACGAACTGGCCCTGCCCTCCAAATATGTGAA
CTTCCTGTACCTGGCCAGCCACTATGAGAAGCTGAAGGGCTCCCCCGAGGATAAT

GAGCAGAAACAGCTGTTTGTGGAACAGCACAAGCACTACCTGGACGAGATCATCG
AGCAGATCAGCGAGTTCTCCAAGAGAGTGATCCTGGCCGACGCTAATCTGGACAA
AGTGCTGTCCGCCTACAACAAGCACCGGGATAAGCCCATCAGAGAGCAGGCCGA
GAATATCATCCACCTGTTTACCCTGACCAATCTGGGAGCCCCTCGGGCCTTCAAGT
ACTTTGACACCACCATCGACCGGAAGGTGTACCGGAGCACCAAAGAGGTGCTGGA
CGCCACCCTGATCCACCAGAGCATCACCGGCCTGTACGAGACACGGATCGACCTG
TCTCAGCTGGGAGGTGACTCTGGCGGCTCAAAAAGAACCGCCGACGGCAGCGAA
TTCGAGCCCAAGAAGAAGAGGAAAGTCTAA

**Supplementary Table 1. AAV expression in cardiomyocytes by region and in other cell types of the left ventricle.** The fraction of cardiomyocytes within the LVs, RVs, LAs, and RAs from 4 male SvEv mice that express ABE transcripts following one dose of AAV treatment. In LVs, the expression transcripts found in additional cell types is quantified.

**Supplementary Table 2. Mouse serum analyses.** Markers for liver toxicity are quantified from serum obtained after treating SvEV (one dose; n=3 males) and SvEv/S4 (two doses; n=3 males) mice with dual-AAV9 carrying ABE. Serum data are shown for SvEv/S4 mice treated with low (n=1 male, 2 females), medium (n=3 females) and high (n=2 females) doses of Cas9KD. For comparison serum from SvEv WT (n=3 males) and untreated 403 SvEV (n=3 males) are shown.

**Supplementary Table 3. RNAseq of untreated and base edited HCM model mice.** The mean expression ±SD of genes related to hypertrophy, the sarcomere, the cytoskeleton, calcium signaling, the extracellular matrix, and metabolism are quantified in untreated R403Q-129SvEv/S4 mice and those treated with two doses of ABE.

**Supplementary Table 4. Summary of off-target sites 1-16.** The genomic coordinates, alignment to the target site, nearest genes, and primers used to amplify each of the 16 tested off-target sites are provided.

**Supplementary Table 5. Genomic off-target sites nominated by CIRCLE-seq.** A list of all off-target coordinates and alignments to the mm10 mouse genome nominated by CIRCLE-seq.
