# Supplementary material for: Efficient in vivo genome editing prevents hypertrophic cardiomyopathy in mice
Source: Nat Med. 2023 Feb 16;29(2):412–21. doi: 10.1038/s41591-022-02190-7 (PMC9941048; doi:10.1038/s41591-022-02190-7)
Supplement: Supplementary file 2 — Reporting Summary [file 41591_2022_2190_MOESM2_ESM.pdf]

nature portfolio

Corresponding author(s): Christine E. Seidman

Last updated by author(s): Dec 13, 2022

Reporting Summary

Nature Portfolio wishes to improve the reproducibility of the work that we publish. This form provides structure for consistency and transparency in reporting. For further information on Nature Portfolio policies, see our Editorial Policies and the Editorial Policy Checklist.

Statistics

For all statistical analyses, confirm that the following items are present in the figure legend, table legend, main text, or Methods section.

n/a	Confirmed	
☐	☒	The exact sample size (*n*) for each experimental group/condition, given as a discrete number and unit of measurement
☐	☒	A statement on whether measurements were taken from distinct samples or whether the same sample was measured repeatedly
☐	☒	The statistical test(s) used AND whether they are one- or two-sided<br>*Only common tests should be described solely by name; describe more complex techniques in the Methods section.*
☒	☐	A description of all covariates tested
☐	☒	A description of any assumptions or corrections, such as tests of normality and adjustment for multiple comparisons
☒	☐	A full description of the statistical parameters including central tendency (e.g. means) or other basic estimates (e.g. regression coefficient) AND variation (e.g. standard deviation) or associated estimates of uncertainty (e.g. confidence intervals)
☐	☒	For null hypothesis testing, the test statistic (e.g. *F*, *t*, *r*) with confidence intervals, effect sizes, degrees of freedom and *P* value noted<br>*Give P values as exact values whenever suitable.*
☒	☐	For Bayesian analysis, information on the choice of priors and Markov chain Monte Carlo settings
☒	☐	For hierarchical and complex designs, identification of the appropriate level for tests and full reporting of outcomes
☒	☐	Estimates of effect sizes (e.g. Cohen's *d*, Pearson's *r*), indicating how they were calculated

Our web collection on statistics for biologists contains articles on many of the points above.

Software and code

Policy information about availability of computer code

Data collection	No software was used
Data analysis	We used open-source CIRCLE-seq analysis software with the default recommended parameters (https://github.com/tsailabSJ/circleseq). For single nuclear RNA sequences we aligned reads using Cell Ranger (v.3.0.1). Editing efficiencies (%) was quantified by CRISPResso2 (https://github.com/pinellolab/CRISPResso2). This information is also stated in the main text / figure legend and in Supporting Information (Methods).

For manuscripts utilizing custom algorithms or software that are central to the research but not yet described in published literature, software must be made available to editors and reviewers. We strongly encourage code deposition in a community repository (e.g. GitHub). See the Nature Portfolio guidelines for submitting code & software for further information.

Data

Policy information about availability of data

All manuscripts must include a data availability statement. This statement should provide the following information, where applicable:

- Accession codes, unique identifiers, or web links for publicly available datasets
- A description of any restrictions on data availability
- For clinical datasets or third party data, please ensure that the statement adheres to our policy

The data availability section of in the manuscript states that data is available without restrictions. We also provide websites for publicly available datasets and indicate that sequencing data generated in this study has been deposited into the NCBI GEO database and accession numbers are provided.

Human research participants

Policy information about studies involving human research participants and Sex and Gender in Research.

Reporting on sex and gender	N/A
Population characteristics	N/A
Recruitment	N/A
Ethics oversight	N/A

Note that full information on the approval of the study protocol must also be provided in the manuscript.

Field-specific reporting

Please select the one below that is the best fit for your research. If you are not sure, read the appropriate sections before making your selection.

☒ Life sciences ☐ Behavioural & social sciences ☐ Ecological, evolutionary & environmental sciences

For a reference copy of the document with all sections, see nature.com/documents/nr-reporting-summary-flat.pdf

Life sciences study design

All studies must disclose on these points even when the disclosure is negative.

Sample size	At least 6 mice were per treatment group were studied as this sample size provided statistical power to assess treatment effects, based on prior studies that demonstrated hypertrophy in all untreated male R403Q-SvEv mice and all male and female R403-SvEV/S4 F1 mice.
Data exclusions	No data are excluded from any analyses
Replication	All mouse experiments were replicated in randomly selected mice and data from all mice are shown. Circle-seq studies were performed twice to replicate initial findings.
Randomization	Mice were randomly allocated to groups.
Blinding	Investigators were blinded to group allocation of mice during data collection and analyses. Blinding was not possible for Circle-seq analyses.

Reporting for specific materials, systems and methods

We require information from authors about some types of materials, experimental systems and methods used in many studies. Here, indicate whether each material, system or method listed is relevant to your study. If you are not sure if a list item applies to your research, read the appropriate section before selecting a response.

Materials & experimental systems

n/a	Involved in the study	
☒	☐	Antibodies
☐	☒	Eukaryotic cell lines
☒	☐	Palaeontology and archaeology
☐	☒	Animals and other organisms
☒	☐	Clinical data
☒	☐	Dual use research of concern

Methods

n/a	Involved in the study	
☒	☐	ChIP-seq
☒	☐	Flow cytometry
☒	☐	MRI-based neuroimaging

Eukaryotic cell lines

Policy information about cell lines and Sex and Gender in Research

Cell line source(s)	HEK293T cells are female. These were obtained from Lonza (https://www/lonza.com)
Authentication	Authentication of HEK293T cells was not performed.

Mycoplasma contamination	Mycoplasma contamination status was not authenticated
Commonly misidentified lines (See ICLAC register)	None

Animals and other research organisms

Policy information about studies involving animals; ARRIVE guidelines recommended for reporting animal research, and Sex and Gender in Research

Laboratory animals	We studied Mus musculus, 129 SvEv and Mus musculus,129 SvEv / S4 F1 hybrids. Mice were randomized into treatment group at ages 10-13 days and study through to 34 weeks of age.
Wild animals	No wild animals were used.
Reporting on sex	We studied only male R403Q-SvEV mice, as females do not develop hypertrophy. All R403Q-SvEv/S4 F1 mice develop hypertrophy and we studied both male and female mice.
Field-collected samples	No field-collected samples were used in this study.
Ethics oversight	All mice were maintained and studied using protocols approved by the Animal Care and Use Committee of Harvard Medical School.

Note that full information on the approval of the study protocol must also be provided in the manuscript.
